# MicroRNA-379-5p attenuates cancer stem cells and reduces cisplatin resistance in ovarian cancer by regulating RAD18/Polη axis

**DOI:** 10.1038/s41419-025-07430-5

**Published:** 2025-02-27

**Authors:** Devendra Shukla, Sanjay Mishra, Tanima Mandal, Manish Charan, Ajeet Kumar Verma, Md Maqsood Ahamad Khan, Nabanita Chatterjee, Amit Kumar Dixit, Senthil Kumar Ganesan, Ramesh K. Ganju, Amit Kumar Srivastava

**Affiliations:** 1 Cancer Biology & Inflammatory Disorder Division, CSIR-Indian Institute of Chemical Biology, Kolkata, West Bengal India; 2 Academy of Scientific and Innovative Research (AcSIR), Ghaziabad, 201002 Uttar Pradesh India; 3 Department of Pathology, The Ohio State University, Columbus, OH USA; 4 Structural Biology & Bioinformatics Division, CSIR-Indian Institute of Chemical Biology, Kolkata, West Bengal India; 5 Chittaranjan National Cancer Institute, Kolkata, India; 6 CCRAS-Central Ayurveda Research Institute, Kolkata, India

**Keywords:** Ovarian cancer, Ovarian cancer, Cancer stem cells

## Abstract

Ovarian cancer (OC) is an aggressive malignancy of the female reproductive organs, associated with a low 5-year survival rate. Emerging evidence suggests the pivotal role of microRNAs (miRNAs) in regulating chemoresistance and metastasis in OC, primarily through cancer stem cells (CSCs), also known as cancer stem-like cells (CSLCs). Herein, we demonstrate that miR-379-5p is downregulated in several OC cell populations including both cell lines and patient tumor samples. Furthermore, overexpression of miR-379-5p effectively inhibits CSCs and counteracts cisplatin-induced expansion of CSCs. Further mechanistic investigations identify RAD18, a DNA repair protein involved in translesion DNA synthesis (TLS), as a direct target of miR-379-5p. Moreover, a negative correlation between miR-379-5p and RAD18 expression is observed in ovarian CSCs isolated from OC patients. The downregulation of RAD18 inhibits stem-like phenotypes and enhances the sensitivity of ovarian CSCs to cisplatin treatment. Importantly, miR-379-5p-mediated inhibition of RAD18 prevents the repair synthesis in CSCs by promoting the accumulation of DNA damage. In vivo studies further reveal that miR-379-5p enhances DNA damage, which, in turn, inhibits tumor cell proliferation in athymic nude mice. Remarkably, targeting of RAD18 by miR-379-5p prevents monoubiquitination of proliferating cell nuclear antigen (PCNA), resulting in reduced DNA Polymerase η (a TLS polymerase that helps to bypass DNA lesions) recruitment to lesion sites. In the absence of Polη, the persisting DNA lesions cause activation of cell cycle arrest and apoptosis pathway in CSCs. Therefore, our findings unveil a novel mechanism whereby miR-379-5p overexpression curtails CSCs by modulating the RAD18/Polη axis.

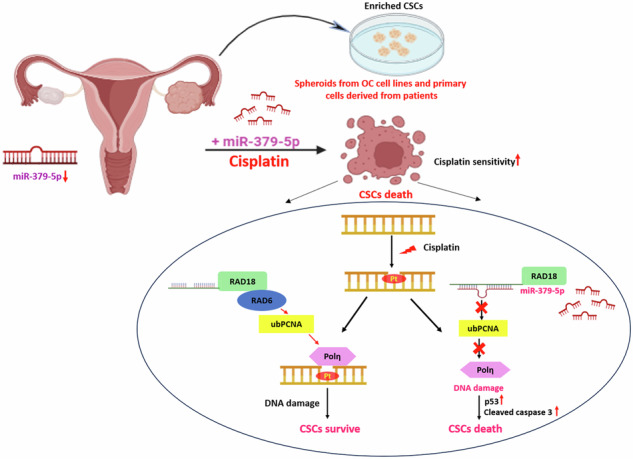

## Introduction

Ovarian cancer (OC) is the leading contributor to gynecological cancer-related fatalities in women [1], with its 5-year survival rate remaining alarmingly low, largely owing to tumor recurrence and chemoresistance [2]. Recent studies have suggested the potential role of cancer stem cells (CSCs), also known as cancer stem-like cells, in chemoresistance, tumor progression, and tumor relapse in cancer patients [3].

CSCs are a specific group of cells within tumors that have unique characteristics, including a higher ability to form tumors, greater self-renewal capacity, and increased heterogeneity [2, 3]. These cells are resistant to standard chemotherapy drugs, such as cisplatin, which work by causing DNA damage in cancer cells. This resistance makes CSCs particularly difficult to eliminate [4]. Their enhanced capacity to repair DNA damage protects them from chemotherapy-induced cell death, leading to an expansion of CSCs after treatment and contributing to tumor relapse [5]. As a result, developing novel strategies to target CSCs is crucial.

MicroRNAs (miRNAs) have been linked to the regulation of most physiological, developmental, and pathological processes, including stem cell regulation [6]. Recent studies have highlighted the involvement of miRNAs in CSC regulation, particularly their impact on self-renewal and chemoresistance [7]. Of particular interest, miR-379-5p has demonstrated its ability to inhibit tumor proliferation in various cancer types, including lung [8], breast [9], cervical [10], and others [11]. However, the ability of miR-379-5p to inhibit tumor proliferation in OC remains uncharacterized. Moreover, the precise molecular mechanisms by which miR-379-5p regulates stemness properties in OC remain elusive.

Translesion synthesis (TLS) is a DNA damage tolerance mechanism that allows cancer cells to bypass DNA lesions instead of repairing them, leading to an accumulation of mutations and increased the risk of cancer development [12]. TLS is a crucial process that protects the CSCs from the harmful effects of DNA-damaging drugs [13, 14]. This process is mediated by monoubiquitination of sliding clamp PCNA by ubiquitin E3 ligase, RAD18, and its corresponding E2 ubiquitin ligase RAD6. The monoubiquitinated-PCNA subsequently recruits the specialized TLS polymerase Polη, which facilitates DNA damage bypass in response to DNA-damaging drugs [15]. RAD18 is a key component of the TLS pathway, with numerous studies providing substantial proof of its role in cancer metastasis and chemoresistance [16–18]. Therefore, RAD18 presents a potential target for overcoming chemoresistance associated with heightened TLS activity in CSCs.

In this study, we elucidate a potential mechanism by which the overexpression of miR-379-5p restrains CSCs and impedes the expansion of cisplatin-induced CSCs through the negative regulation of RAD18 and the TLS pathway. This discovery presents a novel approach for eliminating CSCs and reducing chemoresistance in OC.

## Materials and methods

### Cell culture

OVCAR3, SKOV3, and OV2008 cells were received as gifts from Dr. Qi-En Wang at The Ohio State University Comprehensive Cancer Center. The cells were cultured in RPMI medium supplemented with 10% fetal bovine serum (FBS) and 0.5% penicillin-streptomycin (Pen/Strep). Previous reports have shown that OC cells cultured in spheroids exhibit enriched stem cell-like characteristics [19]. Therefore, to enrich for CSCs, the cells were maintained in DMEM/F12 KnockOut medium supplemented with 10 ng/mL basic fibroblast growth factor, 20 ng/mL epidermal growth factor, and 20% serum replacement. The cells were cultured in ultra-low attachment plates and incubated at 37 °C in a humidified atmosphere with 5% CO_2_. Mycoplasma contamination was assessed prior to the experiments using the MycoAlert detection kit. A complete list of all reagents used in this study is provided in Supplementary Table S1.

### Isolation and culture of tumor cells from freshly excised primary human ovarian tumor tissue

Human ovarian tumors from high-grade serous ovarian cancer (HGSOC) patients were received from Chittaranjan National Cancer Institute (CNCI), Kolkata. All experiments involving human samples were performed at CNCI, Kolkata. Before starting the study, mandatory institutional human ethical clearance was taken (Ref No. CNCI-IEC-40104). Patients were informed about the study’s objectives, and their written consent was obtained. The demographic details of the patients are given in Supplementary Table S5. Samples from four HGSOC patients were used for the analysis. The tumor tissue was minced with a sterile scalpel and was washed three times with 1X PBS. The minced tissue was then treated with collagenase type IV and incubated for 2 h at 37 °C on a shaker. Collagenase activity was blocked by adding media-containing FBS. The solution was then passed through a 40-micron fine mesh, and the filtrate was centrifuged at 300 g for 30 min. The pellet was treated with RBC lysis buffer, washed three times with PBS, and the cells were maintained in both adherent culture (bulk tumor cells) and spheroid culture (enriched CSCs), as mentioned previously.

### Plasmids, miRNA, siRNA, cell transfection, and establishment of stable cell lines

The miR-379-5p mirVana mimic and miRControl were procured from Thermo Fisher Scientific. siRNA trageting RAD18 E3 Ubiquitin (siRAD18) (5′-CCUUCCAAGGAAAGAUUGA-3′) was purchased from Eurogentec. The shMIMIC miR-379-5p lentivirus was procured from Abm (Cat no. mh40575), and the shRAD18 pPB[shRNA]-Puro-U6-hRAD18 (Cat. No. VB200201-100uzu) was acquired from VectorBuilder USA. A small fragment of RAD18 3′-UTR, containing miR-379-5p binding/seed sequence (both wild type and mutant type) was inserted into psiCHECK2 vector at the multiple cloning site (MCS), where RAD18 3′-UTR was placed under the Renilla luciferase gene (Primer sequences are presented in table form in Supplementary Table S3). All miRNA, siRNA, and plasmids were transfected using Lipofectamine 2000.

### Cell sorting and flow cytometry analysis

The percentage of CSCs present within the bulk tumor cells (adherent cells) was determined through flow cytometric analysis using anti-CD117-PE and anti-CD44-FITC (Miltenyi Biotec) antibodies, along with the appropriate isotype controls. Briefly, the cells were stained with anti-CD117-PE and anti-CD44-FITC antibodies and incubated on ice for 30 min in the dark at 4 °C. After incubation, the cells were rinsed with cold 1X PBS before being resuspended in 250 μl of 2% bovine serum albumin (BSA) for flow cytometry analysis using the BD LSRFortessa.

### Live-dead analysis through flow cytometry

CSCs were grown in 60-mm dishes at a density of 0.2 × 10^6^ in spheroid culture, and subsequently transfected with miR-379-5p or siRAD18, along with their respective controls. The control and transfected cells were treated with cisplatin for 12 h, with PBS used as the vehicle control. After 12 h, the spheroids were trypsinized for 5 min and then gently pipetted for single-cell suspension. The suspensions were centrifuged, the medium was decanted, and the cells were washed with 1X PBS. The cells were stained with propidium iodide (5 µg/mL) and analyzed using BD LSRFortessa. The data were reanalyzed by FlowJo software.

### Real-time-PCR analysis

Total RNA was extracted using Trizol, and cDNA synthesis was performed using the cDNA Reverse Transcription kit. TaqMan Universal PCR Master mix and PowerUp™ SYBR™ Green Master Mix were used to perform qRT-PCR analysis. A list of primers is provided in Supplementary Table S2.

### γH2AX flow cytometry analysis

CSCs were cultured at a density of 0.5 × 10^6^ cells per well in ultra-low attachment dishes and grown for 7 days. The cells were transfected with either miRControl, miR-379-5p OE (overexpression), or miR-379-5p OE +RAD18 OE. After 24 h, the cells were treated with either 10 µM cisplatin or PBS for 12 h. Following treatment, the media was replaced, and the cisplatin-treated cells were allowed to recover for 3 h and 6 h, respectively, for DNA repair kinetics assay. The cells were collected, washed with PBS, and suspended in a 50 µl of 2% BSA solution. Subsequently, the cells were stained with antibodies and incubated at room temperature for 30 min in the dark. The cells were then washed and resuspended in 2% BSA, and analyzed using BD LSRFortessa.

### Confocal imaging

The cells were transfected with either the miR-379-5p mimic or miRControl. A total of 0.5 × 10^5^ cells were seeded on coverslips and incubated overnight to allow attachment. After the cells adhered, they were treated with either 10 µM cisplatin or PBS for 12 h. Subsequently, the cells were fixed using 4% ice-cold formaldehyde for 30 min. To block non-specific binding, the cells were incubated with 5% BSA on a shaker at 4 °C for 1 h. The cells were then washed and exposed to the primary antibody. The dishes were incubated at 4 °C on a shaker overnight. Following this, cells were then washed with 1X PBS and incubated with a secondary anti-Rabbit AlexaFluor488 antibody (AB150077, Abcam) at room temperature for 1 h, with the dishes placed on a shaker. The cells were washed three times with PBS for 5 min each. Afterward, the cells were counterstained with hoechst (10 μg/mL) and incubated for 5 min at room temperature on a shaker. Subsequently, a PBS wash was performed three times. The coverslips were mounted on grease-free slides, and the cells were examined under a confocal microscope.

### HPRT assay

Mutagenesis in OC cells was assessed using the Hypoxanthine-guanine phosphoribosyl transferase (HPRT) assay as described earlier [20]. The miR-379-5p-proficient and miRControl cells were treated with 10 µM cisplatin for 24 h, followed by selection for 6-thioguanine (6-TG) resistance over a 10-day period. The cells were then fixed in ice-cold formaldehyde (4%) and stained with 0.5% methylene blue. 6-TG-resistant colonies were counted to determine the mutation frequency.

### Xenograft transplantation and tumor study

Athymic nude NRU-Foxn1^nu^ mice, aged 6–8 weeks, female, with a body weight of 20–23 g, were obtained from the OSU TVSR facility. Animal care and experiments were conducted following the institutional guidelines and with the approval of the Institutional Animal Care and Use Committee at OSU. For the xenograft model, 50 μl of RPMI containing 2 × 10^6^ cells of miR-379-5p-proficient or RAD18-deficient OVCAR3 cells were mixed in a 1:1 ratio with Matrigel and injected subcutaneously into flanks of nude mice. The mice were randomly segregated into four groups (*n* = 5). After a quarantine period of 7 days, the mice were administered with an intraperitoneal injection of cisplatin (100 µl, 5 mg/kg b.wt.) once weekly for 3 weeks. The mice were then euthanized, and tumors were excised. The tumors obtained were embedded in paraffin, and tissue sections (4 μM) were prepared on slides for histopathological study.

### Immunohistochemistry analysis

Slides with paraffin-embedded tissue sections were incubated at 60 °C for 30–40 min in a hot air oven. Following this step, the slides were rehydrated by sequentially dipping them in xylene (twice), followed by 100%, 90%, 70%, and 50% isopropanol, with each step lasting 5 min. The slides were then washed in a wash buffer (1X PBS with 0.1% Triton X-100) for 2 min, and this washing step was repeated two more times. Afterward, the slides were dipped in 10 mM tri-sodium citrate buffer and boiled for 30 min in the microwave at full wattage for antigen retrieval. The staining was performed using a Rabbit Specific HRP/DAB Detection kit as per the manufacturer’s protocol. The tissue samples were counterstained with hematoxylin, and then dehydrated by sequentially dipping the slides into 50%, 70%, 90%, and 100% alcohol, followed by xylene. Finally, the slides were observed under an inverted microscope at Central Instrument facility, CSIR-IICB Kolkata.

### Immunoprecipitation

OVCAR3 cells were cultured in a 100 mm dish until reaching 80% confluency. Subsequently, the cells were harvested, and whole cell lysate was prepared in RIPA buffer containing a 1× protease inhibitor. The lysates were then sonicated and centrifuged at 10,000 rpm for 10-15 min to obtain supernatant. The lysates were pre-cleared by incubating them with protein A agarose beads (Cat no. 20333, Thermo Fisher) for 1 h at 4 °C. Following this, an equivalent amount of lysate was then incubated overnight at 4 °C with the specific antibodies of interest, on a rotor set to 700 rpm. The immunocomplexes were captured by binding to protein A beads with gentle rotation for 4 h at 4 °C. The beads were collected by centrifuging at 10,000 rpm for 10 min and washed with an excess of RIPA buffer. Proteins were eluted by boiling the beads in a 2× protein gel loading buffer. The eluted proteins were then used for SDS-PAGE and western blotting. Whole cell lysate was used as a reference input sample.

### Luciferase reporter assay

SKOV3 cells were plated in a 96-well plate at a density of 0.5 × 10^5^ cells and incubated for 24 h. The cells were then co-transfected with 200 ng of psiCHECK2-RAD18 3′-UTR wild type or psiCHECK2-RAD18 3′-UTR mutant and 20 μmol/L of miR-379-5p mimic. After 48 h, the cells were collected, and the firefly and Renilla luciferase activities were determined using the Dual-Luciferase Reporter Assay System (E1910, Promega). Further, Renilla activity was normalized to firefly activity to access the consequence of miR-379-5p overexpression on RAD18 expression.

### Western blotting

The cells were transfected with miRNA or siRNA, along with a control group, and incubated for 48 h. Cell lysis was performed using RIPA buffer. Equal amounts of proteins were separated using SDS-PAGE and transferred to a methanol-activated PVDF membrane. The blots were incubated with specific antibodies overnight at 4 °C. Subsequently, the membrane was incubated with a secondary antibody for 2 h at room temperature. Protein bands were detected using the ECL substrate. Supplementary Table S4 provides the details of the antibodies used. All original blots are included in Supplementary material.

### Protein-protein docking analysis

For the protein interaction analysis, the protein structures of RAD18, Polη, and PCNA were retrieved from the RCSB PDB database (https://www.rcsb.org/). Protein-protein docking was carried out using the HDOCK and ClusPro online web servers, and the predicted binding scores were determined. The docking results were visualized using PyMOL and Chimera software. Docking scores obtained from the ClusPro analysis are presented in tabular form in Supplementary Table S7.

### Statistical analysis

Data analysis was performed using a one-way ANOVA and an unpaired Student’s *t*-test. Experiments were performed in triplicate, and the results are expressed as the mean ± SD. Statistical significance was set at a *p* value of <0.05.

## Results

### Bioinformatics prediction reveals that downregulation of miR-379-5p may be necessary for the maintenance and proliferation of ovarian CSCs

**Fig. 1:**
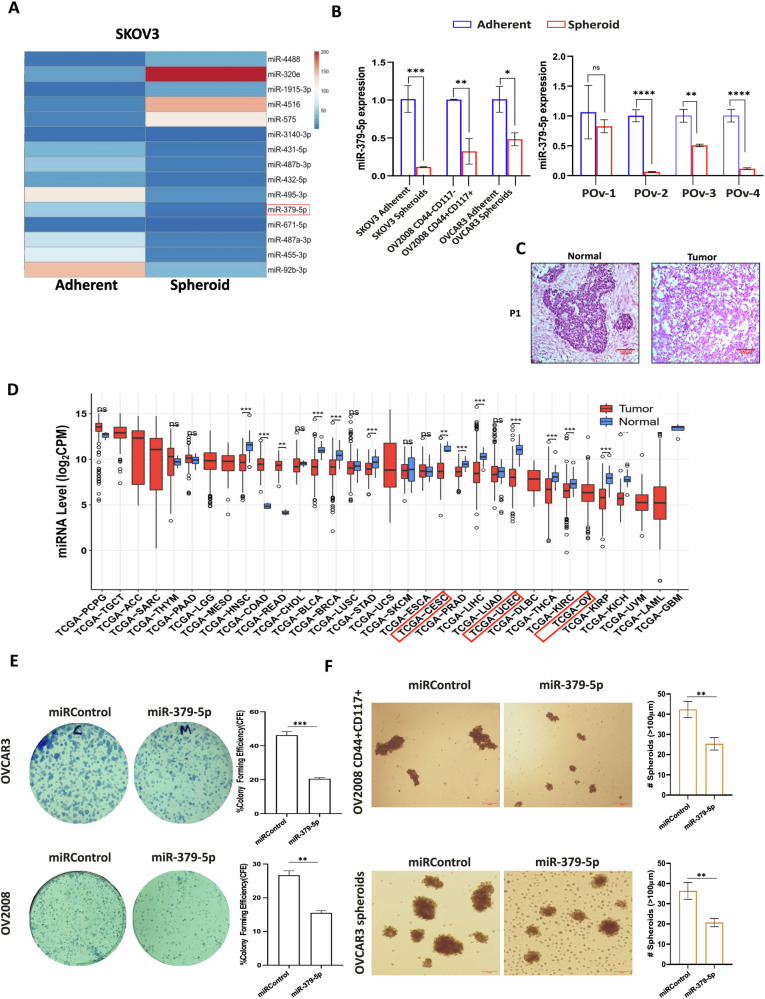
miR-379-5p is downregulated in ovarian CSCs. **A** The GEO dataset GSE107155 was reanalyzed to identify a list of differentially expressed microRNAs in ovarian cancer cell line SKOV3 grown in adherent and spheroid cultures. **B** qPCR analysis showed decreased miR-379-5p expression in spheroids derived from ovarian cancer cell lines and HGSOC patients. **C** Representative H&E of normal tissue and tumor tissue derived from ovarian cancer patients. **D** Pan-cancer expression profiling of miR-379-5p in normal vs tumor samples using CancerMIRNome (Wilcoxon rank-sum test, ***: *P* < 0.001; **: *P* < 0.01; *: *P* < 0.05; ns: *P* > 0.05). **E** Colony formation assay was conducted on control and miR-379-5p-overexpressing OC cells. **F** Representative images show spheroid formation ability in control and miR-379-5p-overexpressing ovarian CSCs (*n* = 3, Bar, SD; Significance levels denoted as ns > 0.05, *P < 0.05, **P < 0.01, ***P < 0.001 and ****P < 0.0001).

We began our study by reanalyzing the differentially expressed microRNAs in OC patients using the GEO Dataset GSE239685 (Supplementary Fig. 1A). Our analysis identified the most significantly downregulated microRNAs in OC tissues compared to normal tissues, with miR-379-5p being notably reduced. Subsequently, we examined the differential expression of microRNAs in OC using the GEO Dataset GSE107155. Among the identified miRNAs, miR-379-5p was again found to be significantly downregulated in CSCs enriched spheroid cultures, as compared to the corresponding epithelial ovarian cancer (EOC) cells grown in adherent cultures (Fig. 1A). Next, we confirmed the downregulation of miR-379-5p in CSCs using quantitative real-time PCR (qRT-PCR) analysis of spheroids derived from three OC cell lines and four high-grade serous ovarian cancer (HGSOC) tumors (Fig. 1B). These findings suggest that the downregulation of miR-379-5p may be associated with the maintenance of stemness properties in ovarian CSCs.

In addition, our in silico pan-cancer analysis revealed that miR-379-5p is downregulated in most gynecological malignancies, including cervical, uterine, and ovarian cancer (Fig. 1D). However, due to the lack of normal tissue samples for direct comparison with the OC samples in the aforementioned dataset, we validated the downregulation of miR-379-5p in OC tissue relative to normal controls using the GEO dataset GSE239685, indicating its potential role as a tumor suppressor (Supplementary Fig. 1A). Furthermore, colony formation assays demonstrated an antiproliferative effect of miR-379-5p overexpression in OC cells, confirming its tumor-suppressor function (Fig. 1E). Cells overexpressing miR-379-5p also exhibited a significantly reduced capacity to form spheroids (Fig. 1F), supporting its role in regulating CSC enrichment. The effects of miR-379-5p overexpression were further analyzed through cell migration and wound healing assays, which revealed significantly reduced migration and wound healing abilities in OC cells (OVCAR3 and OV2008) compared to control groups (Supplementary Figs. 1B and 2).

### Overexpression of miR-379-5p inhibits cisplatin-induced CSC expansion in both in vitro and in vivo pre-clinical models

**Fig. 2:**
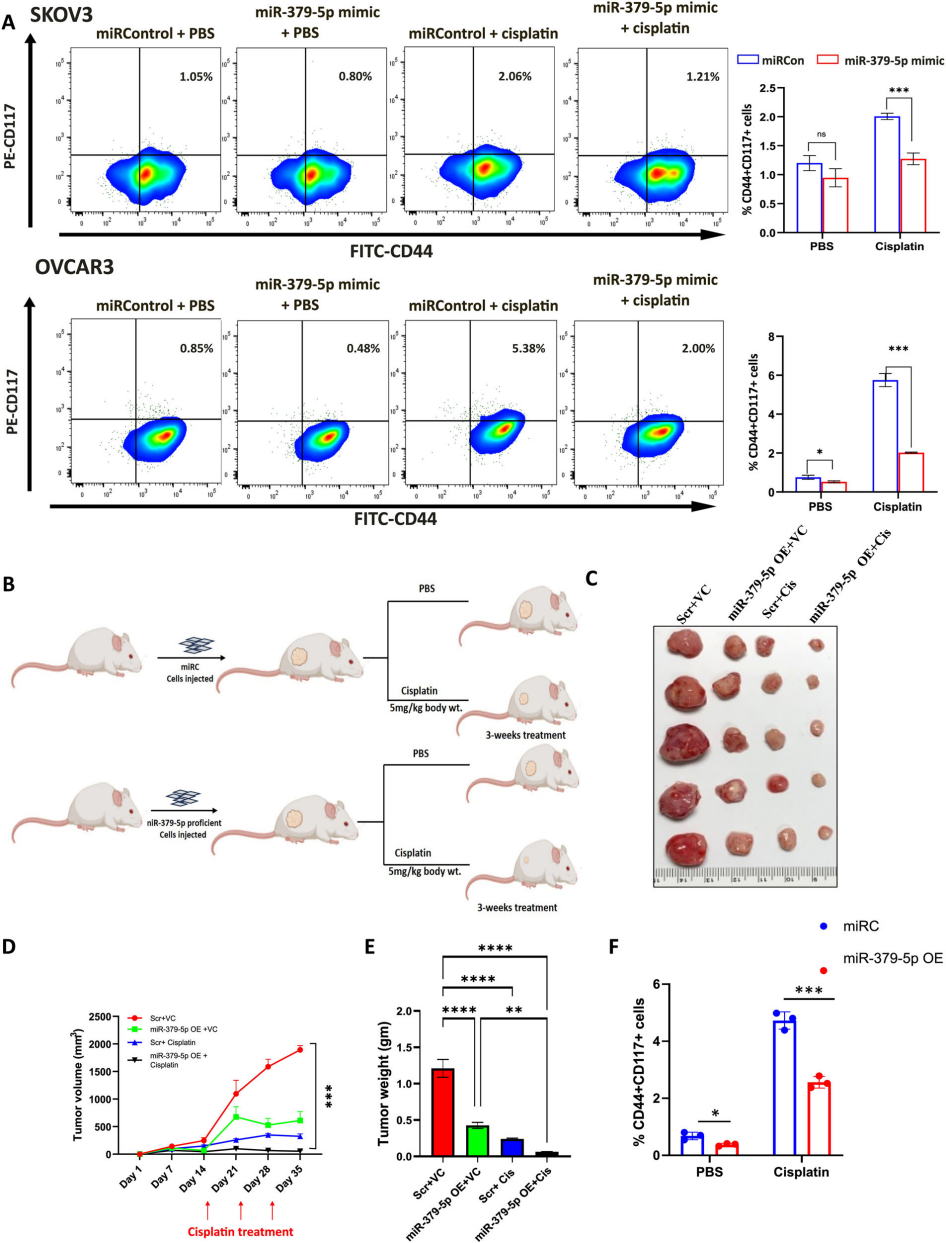
miR-379-5p downregulation is required for CSCs maintenance. **A** Overexpression of miR-379-5p reduces the enrichment of CD44 + CD117+ cell populations within SKOV3 and OVCAR3 bulk cells following cisplatin treatment. CD44 + CD117+ cells were identified using flow cytometry, and the accompanying graph shows the percentage of CD44 + CD117+ cell enrichment. **B** Athymic nude mice (*n* = 5) were injected with either miRControl or miR-379-5p-proficient OVCAR3 cells. Once the tumor diameter reached around 0.5 cm, both groups of mice received weekly treatments with PBS or cisplatin (5 mg/Kg body weight) for 3 weeks. **C** Representative images of the tumors 3 weeks after the initial drug treatment. **D** Quantification of the time-dependent progression in tumor volume, measured with calipers. **E** After the mice were sacrificed, tumor weights were determined in grams. **F** The graph shows that xenograft cells with miR-379-5p overexpression exhibit a reduced enrichment of CD44 + CD117+ cell populations in response to cisplatin treatment (*n* = 3, Bar, SD; Significance levels indicated as *P < 0.05, **P < 0.01, ***P < 0.001 and ****P < 0.0001).

The bulk tumor (adherent) cells contain a subpopulation of CSCs within them. While most platinum-based drugs effectively target bulk tumor cells, they are often ineffective against CSCs, leading to their enrichment. To investigate the role of miR-379-5p in CSC enrichment in OC, we overexpressed miR-379-5p in adherent cells and subsequently treated them with cisplatin. Overexpression of miR-379-5p in EOC cell lines (SKOV3 and OVCAR3) effectively blocked cisplatin-induced CSC expansion by reducing the proportion of CSCs, as defined by the CD44 + CD117+ markers [21] (Fig. 2A). This finding was corroborated in an in vivo xenograft model treated with cisplatin (Fig. 2B). Tumor xenografts overexpressing miR-379-5p exhibited a more pronounced regression in tumor volume and decreased growth compared to tumors lacking miR-379-5p (Fig. 2C–E).

Additionally, flow cytometry analysis revealed that in vivo cisplatin treatment enriched CSCs in control xenograft cells but not in miR-379-5p-proficient tumor xenografts. Notably, tumors derived from miR-379-5p-proficient cells contained significantly fewer CD44 + CD117+ cells (Fig. 2F). Together, these findings underscore the critical role of miR-379-5p in suppressing tumor growth and proliferation in OC by inhibiting the CSC subpopulation.

### Overexpression of miR-379-5p reduces stemness in ovarian CSCs and sensitizes them to cisplatin treatment

**Fig. 3:**
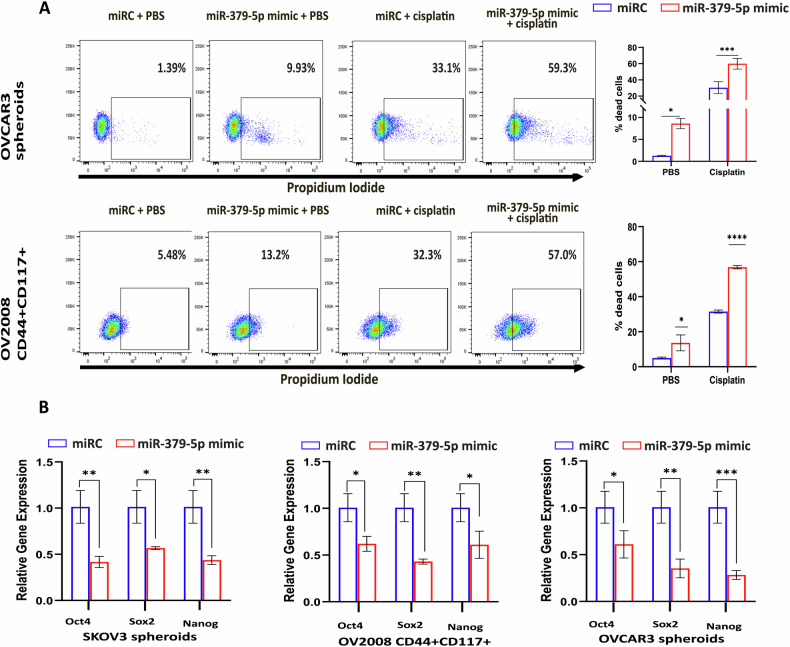
miR-379-5p enhances the sensitivity of CSCs to cisplatin while decreasing the expression of genes associated with stemness. **A** Propidium iodide (PI) live-dead assay on OVCAR3 spheroid cells and OV2008 CD44 + CD117+ cells exhibit higher cytotoxicity in cells overexpressing miR-379-5p upon treatment with cisplatin. The accompanying chart illustrates the percentage of cell death. **B** In SKOV3 spheroids, OV2008 CD44 + CD117+ cells, and OVCAR3 spheroids, the overexpression of miR-379-5p significantly diminishes the expression of stem cell marker genes such as oct4, sox2, and nanog (*n* = 3, Bar, SD; Significance levels are denoted as *P < 0.05, **P < 0.01, ***P < 0.001, and ****P < 0.0001).

As previously mentioned, CSCs are more resistant to cisplatin treatment compared to adherent tumor cells [4]. To investigate the functional role of miR-379-5p in cisplatin resistance in CSCs, we enriched the CSC population in OC cell lines (SKOV3, OV2008, and OVCAR3) through spheroid culture (Supplementary Fig. 3A). The spheroids exhibited higher expression levels of stem cell markers (Sox2, Oct4, and Nanog) (Supplementary Fig. 3B, C) and lower accumulation of reactive oxygen species (ROS) compared to the adherent EOC cells (Supplementary Fig. 3D, E). Additionally, flow cytometry and CFDA/PI live-dead imaging analyses indicated that overexpression of miR-379-5p enhanced the susceptibility of ovarian CSCs to cisplatin treatment (Fig. 3A; Supplementary Fig. 4). Furthermore, overexpressing miR-379-5p reduced the expression of CSC markers (Sox2, Oct4, and Nanog) in SKOV3, OV2008, and OVCAR3 spheroids (Fig. 3B). In summary, these results suggest that miR-379-5p overexpression facilitates the depletion of CSCs following cisplatin treatment.

### RAD18 is highly expressed in ovarian CSCs and is a direct target for miR-379-5p

**Fig. 4:**
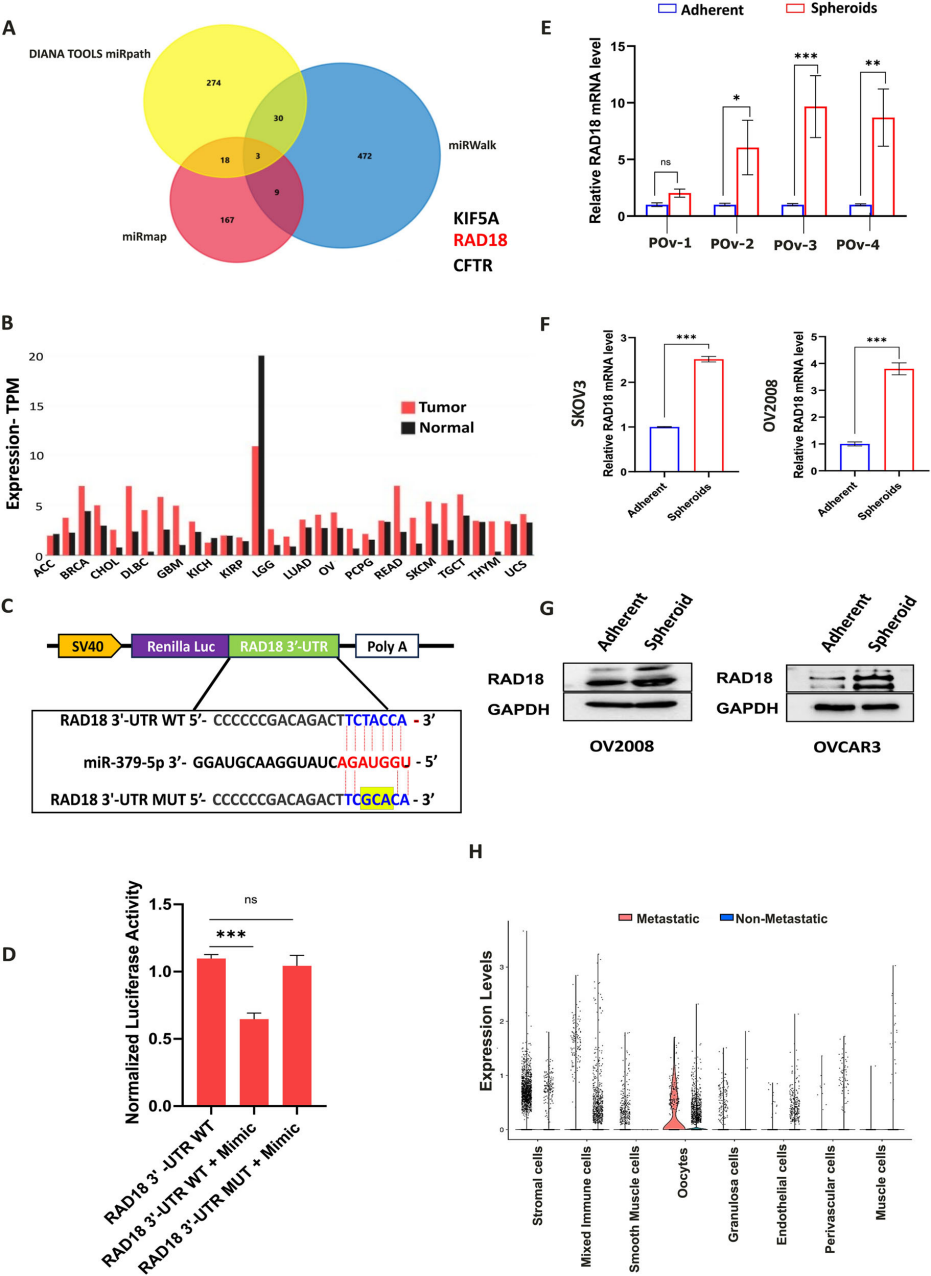
RAD18, a direct target for miR-379-5p. **A** The Venn diagram illustrates three common targets (KIF5A, RAD18, and CFTR) of miR-379-5p identified from various databases. **B** Pan-cancer expression analysis of RAD18 in tumor vs. normal samples using GEPIA. **C** Schematic diagram illustrating the alignment of miR-379-5p with its wild-type binding site in the Homo sapiens RAD18 3′UTR sequence, along with the corresponding mutated binding site. **D** Results from the dual-luciferase reporter assay reveal the direct interaction between miR-379-5p and RAD18. OVCAR3 cells were transfected with either wild-type RAD18 3′-UTR-psiCHECK2/miR-379-5p or mutated RAD18 3′-UTR-psiCHECK2/miR-379-5p alongside a wild-type 3′-UTR-psiCHECK2/scramble miRControl. Renilla luciferase activity was measured, and normalized to firefly activity. **E** The expression levels of RAD18 in HGSOC patients were determined via qPCR. **F**, **G** RAD18 levels were analyzed in SKOV3 adherent and spheroid cells and OV2008 CD44-CD117- and CD44 + CD117+ cells through qPCR and western blotting. **H** Single-cell RNA analysis demonstrates the variation in RAD18 expression between metastatic and non-metastatic conditions in different cell types (*n* = 3, Bar, SD; Significance levels indicated as *P < 0.05, **P < 0.01, and ***P < 0.001).

To identify the potential direct molecular target responsible for the observed effects of miR-379-5p in ovarian CSCs, we conducted an in silico analysis using multiple miRNA databases, including miRWalk, miRMap, and DIANA Tools miRPath. This analysis identified three common candidate genes, including RAD18 (Fig. 4A). RAD18 is a key component in the TLS pathway and has been associated with the development of chemoresistance in cancer cells [13, 21]. Based on these findings, RAD18 was selected for further mechanistic investigation in this study.

A pan-cancer analysis of RAD18 expression revealed elevated levels in OC tissues compared to normal tissues (Fig. 4B). Sequence alignment analysis revealed a putative binding site for miR-379-5p within the 3′-untranslated region (3′-UTR) of RAD18 (Fig. 4C). To further investigate this, we aimed to characterize the miR-379-5p binding site within the 3′-UTR of RAD18. We cloned the wild-type 3′-UTR of RAD18, including the miR-379-5p binding site, into the psiCHECK2 vector. Additionally, mutant constructs of the 3′-UTR with mutations in the miR-379-5p binding site were generated and cloned into the psiCHECK2 vector (Fig. 4C).

Subsequently, a dual-luciferase assay was performed in OVCAR3 cells using a miR-379-5p mimic along with either the wild-type or mutant 3′-UTR-psiCHECK2 constructs. Our results revealed a significant decrease in luciferase activity in cells transfected with the wild-type 3′-UTR-psiCHECK2/miR-379-5p mimic compared to those transfected with the wild-type 3′-UTR-psiCHECK2/scrambled miRcontrol. In contrast, no change in luciferase activity was observed in OVCAR3 cells transfected with the mutant 3′-UTR-psiCHECK2/miR-379-5p mimic, suggesting that RAD18 is a direct target of miR-379-5p (Fig. 4D).

Moreover, high RAD18 expression was confirmed in ovarian CSCs, particularly in spheroid cultures of SKOV3, OVCAR3, and OV2008 CD44 + CD117+ cells, compared to their adherent counterparts (Fig. 4E–G). Additionally, bioinformatics analysis using TNMplot revealed elevated RAD18 expression across various tumor types, including OC (Supplementary Fig. 5A). Comparison of normal versus tumor tissue also indicated significantly higher RAD18 levels in tumor samples (Supplementary Fig. 5B).

Interestingly, the protein-protein interaction network for RAD18, provided by the STRING database, indicated interactions with other proteins involved in the TLS pathway, including Polη (Supplementary Fig. 5C). Furthermore, single-cell RNA sequencing (scRNA-seq) analysis of publicly available ovarian datasets confirmed the presence of seven distinct ovarian cell types (Supplementary Fig. 6A, B), with endothelial, stromal, and oocyte cell types exhibiting higher RAD18 expression levels (Fig. 4H; Supplementary Fig. 7A, B).

### Silencing RAD18 expression inhibits CSCs and enhances cisplatin efficacy against CSCs

**Fig. 5:**
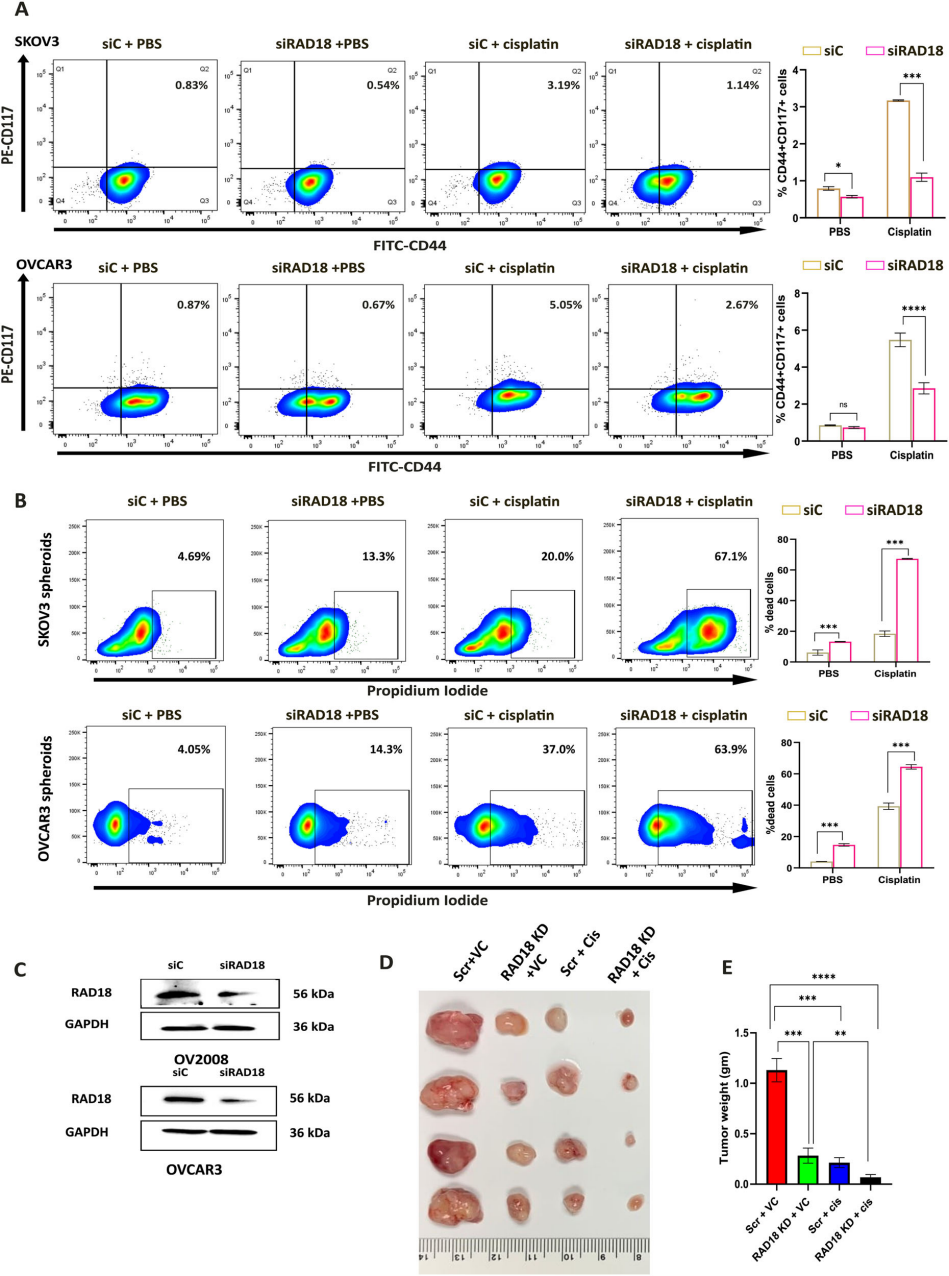
RAD18 expression is associated with CSCs progression. **A** Downregulation of RAD18 reduces the enrichment of CD44 + CD117+ cell populations within SKOV3 and OVCAR3 bulk cells following cisplatin treatment. CD44 + CD117+ cells were identified using flow cytometry, and the accompanying graph shows the percentage of CD44 + CD117+ cell enrichment. **B** The propidium iodide (PI) live-dead assay on SKOV3 and OVCAR3 spheroids showed enhanced cytotoxicity in cells with RAD18 downregulation after cisplatin treatment. **C** Downregulation of RAD18 was confirmed through western blotting in OV2008 and OVCAR3. **D** The downregulation of RAD18 in xenograft transplants resulted in reduced tumor size in athymic nude mice treated with either PBS or cisplatin (5 mg/kg body weight). **E** Tumor weights were measured in grams (gm) at the conclusion of the experiments (Bar, SD; Significance levels indicated as *P < 0.05, **P < 0.01, ***P < 0.001 and ****P < 0.0001).

Having established RAD18 as a direct target of miR-379-5p, we aimed to understand its role in the maintenance of stem-like properties and mediating cisplatin resistance. We first silenced RAD18 expression in EOC cell lines using siRAD18 (Fig. 5C). Following RAD18 silencing, cisplatin treatment was less effective at inducing CSC enrichment, confirming RAD18’s critical role in mediating cisplatin resistance (Fig. 5A). These findings were further validated in CSCs isolated from various EOC cell lines, where RAD18 downregulation resulted in increased cytotoxicity in RAD18-deficient CSCs, as demonstrated by live-dead staining (Fig. 5B). The results were corroborated through live-dead imaging (Supplementary Fig. 8). Additionally, xenograft tumors generated by RAD18-deficient cells exhibited slower regrowth compared to control groups after cisplatin treatment, as indicated by lower tumor burden in RAD18-deficient group (Fig. 5D, E). Taken together, these in vitro and in vivo results emphasize the importance of RAD18 in regulating cisplatin-induced CSCs enrichment.

### miR-379-5p impairs DNA repair in CSCs by modulating RAD18 expression and reduces cisplatin-induced mutagenesis

**Fig. 6:**
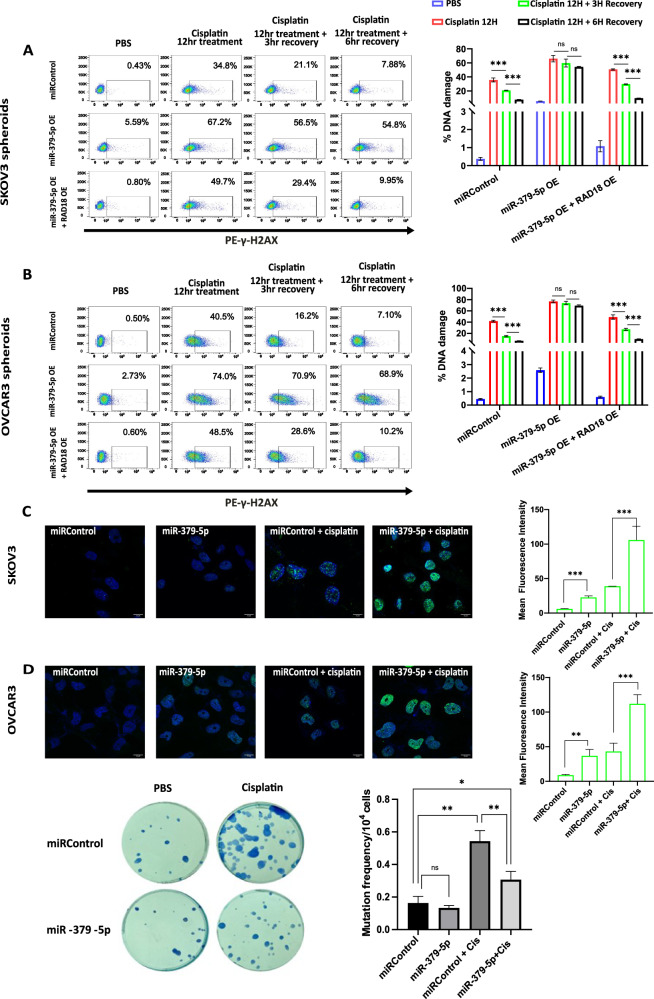
miR-379-5p impairs DNA repair and reduces cisplatin-induced mutagenesis. **A**, **B** Flow cytometry analysis of γ-H2AX, a marker for DNA damage, in SKOV3 spheroids and OVCAR3 spheroids, reveals increased γ-H2AX levels in cells having miR-379-5p overexpression (OE: overexpression); The DNA repair kinetics assay revealed insignificant DNA damage repair in cells with miR-379-5p OE group, compared to the significant repair observed in both the miRControl and miR-379-5p OE + siRAD18 OE groups after 3 h and 6 h of recovery. **C**, **D** Immunofluorescence study of γ-H2AX accumulation in SKOV3 and OVCAR3 adherent cells overexpressing miR-379-5p. The corresponding graph indicates the mean fluorescence intensity (MFI) of γ-H2AX staining. **E** Mutagenesis analysis in OVCAR3 adherent cells with miR-379-5p overexpression revealed reduced mutagenesis in response to cisplatin treatment compared to control cells. The corresponding graph shows the mutation frequency observed in each group (*n* = 3, Bar, SD; Significance levels indicated as *P < 0.05, **P < 0.01, and ***P < 0.001).

CSCs are known to possess enhanced DNA repair capabilities which contribute to chemoresistance [22]. Given that RAD18 is a DNA repair protein involved in translesion DNA synthesis, we hypothesized that miR-379-5p, by targeting RAD18, might impair DNA repair in CSCs. In our study, we observed that the cells overexpressing miR-379-5p showed a higher accumulation of γH2AX upon cisplatin treatment, suggesting greater DNA damage compared to control cells as well as cells overexpressing both miR-379-5p and RAD18. This indicates that miR-379-5p increases the accumulation of DNA damage via inhibiting RAD18 expression. Moreover, miR-379-5p overexpression also impaired the ability of spheroid cells to repair DNA damage. The DNA repair kinetics assay revealed significantly less DNA damage repair following the 3- and 6-h recovery periods in ovarian CSCs overexpressing miR-379-5p, compared to the control group and miR-379-5p OE + RAD18 OE groups (Fig. 6A, B). These results were further corroborated by the γH2AX foci formation assay, which showed higher accumulation of γH2AX in cells overexpressing miR-379-5p compared to the miRControl group after cisplatin treatment (Fig. 6C, D) (Supplementary Fig. 9). Moreover, miR-379-5p overexpression inhibited cisplatin-induced mutagenesis, as evidenced by a decreased number of colonies in the groups overexpressing miR-379-5p, further supporting its role in reducing mutagenic potential (Fig. 6E).

### miR-379-5p-mediated inhibition of DNA repair causes persistent DNA damage which limits proliferation in xenografts

**Fig. 7:**
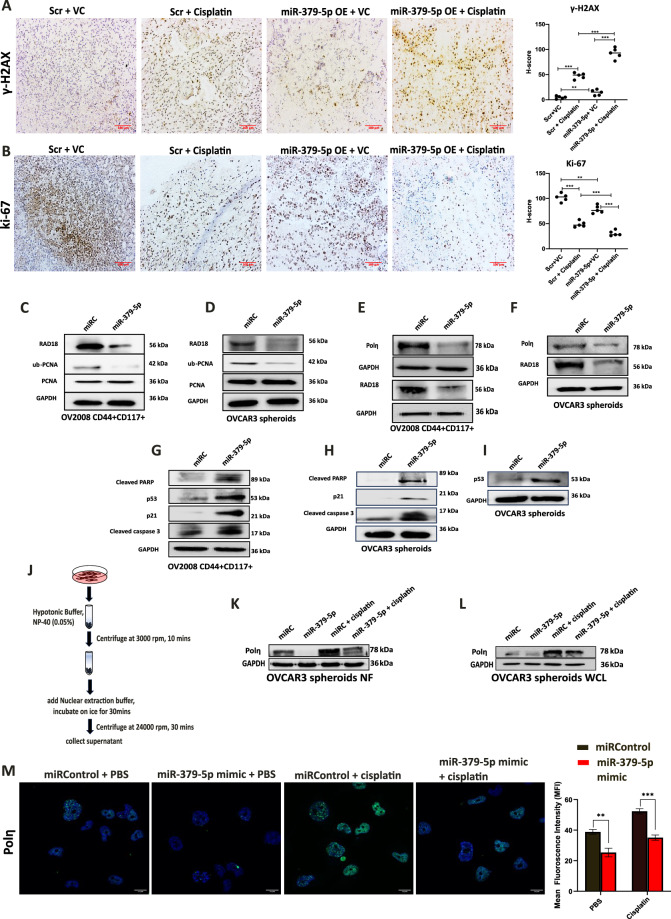
miR-379-5p induces DNA damage, suppresses proliferation in xenografts, and modulates the TLS pathway via targeting RAD18, leading to cell cycle arrest and apoptosis in CSCs. **A** Immunohistochemistry (IHC) staining using γ-H2AX, a marker of DNA damage, in tumor tissues obtained from xenografts. **B** IHC staining employing Ki-67 in tumor tissues derived from xenografts. The corresponding graphs depict the H-scores for each staining. **C**, **D** miR-379-5p overexpression in CSCs inhibits RAD18-mediated monoubiquitination of PCNA in OV2008 CD44 + CD117+ and OVCAR3 spheroids. **E**, **F** The nuclear fraction isolated from miR-379-5p mimic-transfected cells displays reduced Polη and RAD18 expression in OV2008 CD44 + CD117+ and OVCAR3 spheroids. **G**–**I** miR-379-5p overexpression activates apoptotic genes in OV2008 CD44 + CD117+ and OVCAR3 spheroids. **J** The schematic diagram outlines the nuclear fraction isolation method utilized in this study. **K** Polη expression levels in response to PBS or cisplatin treatment in nuclear fractions (NF) isolated from miRC or miR-379-5p-overexpressing OVCAR3 spheroids. **L** Polη expression levels in response to PBS or cisplatin treatment in whole cell lysate (WCL) isolated from miRC or miR-379-5p-overexpressing OVCAR3 spheroids. **M** miR-379-5p mimic prevents the increased Polη expression in response to cisplatin treatment. The corresponding graph indicates the mean fluorescence intensity (MFI) values of Polη immunostaining (*n* = 3, Bar, SD; Significance levels denoted as **P < 0.01, and ***P < 0.001).

CSCs are essential for tumor metastasis and recurrence [23]. After we observed that miR-379-5p inhibits DNA repair *via* RAD18 targeting we sought to investigate the impact of miR-379-5p overexpression in vivo conditions. Our findings revealed that the overexpression of miR-379-5p in ovarian tumor xenografts inhibited tumor cell proliferation and resulted in the accumulation of DNA damage following cisplatin treatment. Immunohistochemistry (IHC) analysis revealed an increased accumulation of γH2AX in tumor tissues overexpressing miR-379-5p, especially following cisplatin treatment (Fig. 7A). Moreover, IHC analysis showed that miR-379-5p overexpression reduced cancer cell proliferation, as evidenced by lower ki-67 (cell proliferation marker) levels in miR-379-5p-overexpressing tissues post-cisplatin treatment (Fig. 7B). Overall, these results demonstrated that miR-379-5p restricts OC growth by impairing the proliferative capacity of tumor cells and enhancing DNA damage in xenografts.

### Targeting of RAD18 by miR-379-5p prevents ub-PCNA-mediated TLS polymerase Polη recruitment to DNA damage sites, inhibiting lesion bypass, and leading to apoptosis in CSCs

Following these observations, we investigated the mechanism through which the miR-379-5p/RAD18 axis regulates stemness and chemoresistance in ovarian CSCs. The RAD6/RAD18 complex monoubiquitinates PCNA, facilitating the recruitment of Polη to the lesion site [24]. Western blot analysis revealed a marked reduction in the expression levels of RAD18 and ubiquitinated PCNA (ub-PCNA) in ovarian CSCs transfected with miR-379-5p mimic (Fig. 7C, D). In the absence of monoubiquitination of PCNA, we anticipated a reduced Polη engagement with DNA lesions. To test this, we isolated nuclear fractions from miR-379-5p-overexpressing cells (Fig. 7J) and found a significantly lower Polη recruitment (Fig. 7E, F). This impairment of Polη recruitment hampers the DNA repair process, resulting in activation of p53-mediated cell cycle arrest and apoptosis, as confirmed by western blotting (Fig. 7G–I). Furthermore, miR-379-5p overexpression significantly restricted cisplatin-induced Polη recruitment to the nucleus in ovarian CSCs (Fig. 7K), as evidenced by a marked reduction in Polη levels in the nuclear fraction isolated from OVCAR3 spheroids. Although Polη expression was also diminished in the whole cell lysate from miR-379-5p-overexpressing OC spheroids following cisplatin treatment, the reduction within the nuclear fraction was more pronounced (Fig. 7L), suggesting that miR-379-5p specifically impedes Polη recruitment to the nucleus in response to DNA damage. This finding was further corroborated by immunofluorescence staining, which revealed decreased Polη localization in the nuclei of OVCAR3 cells overexpressing miR-379-5p upon cisplatin treatment (Fig. 7M).

**Fig. 8:**
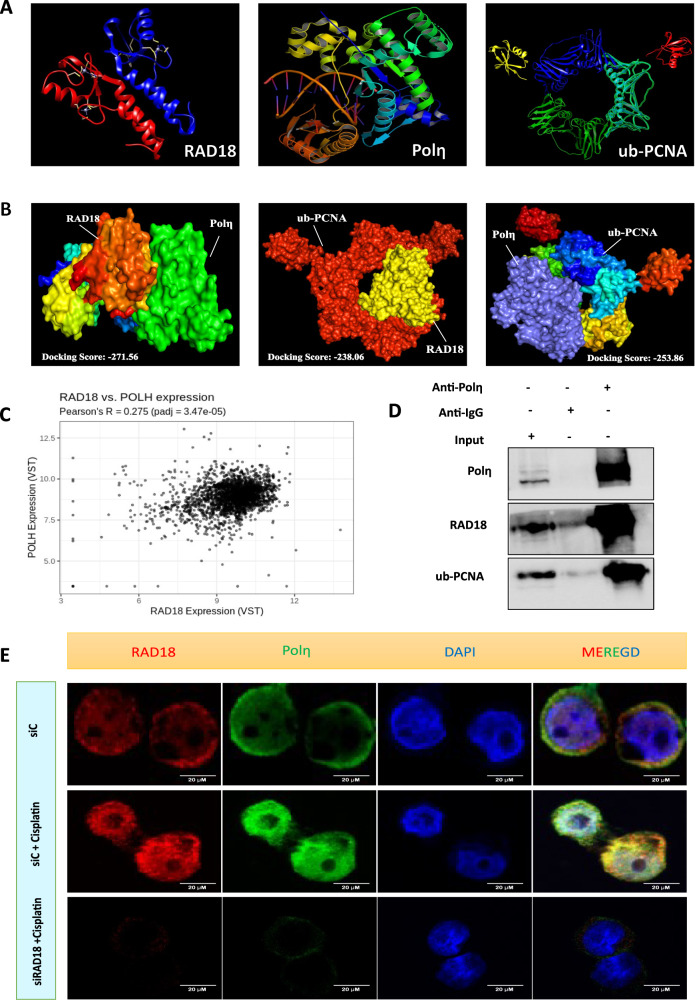
RAD18 facilitates the recruitment of Polη to the DNA damage sites within the nucleus. **A** The ribbon diagram of RAD18, Polη, and ub-PCNA reproduced from PDB. **B** Protein-protein docking for RAD18-Polη, RAD18- ub-PCNA, and Polη-ub-PCNA was performed using HDOCK. **C** A positive correlation between RAD18 and Polη expression in female gynecological malignancies was obtained from the CorrelationAnalyzeR tool. **D** Co-immunoprecipitation of RAD18, ub-PCNA, and Polη was performed using Polη antibody. A representative western blot image validates the interaction between RAD18, ub-PCNA, and Polη. **E** Immunofluorescence staining of OVCAR3 adherent cells with RAD18 and Polη showed that Polη had reduced nuclear localization in the absence of RAD18 following cisplatin treatment.

To understand the interaction among RAD18, Polη, and PCNA, we performed in silico protein-protein docking using the HDOCK and ClusPro web servers (Fig. 8A). In the HDOCK web server, docking was performed between RAD18-Polη, RAD18-PCNA, and Polη-PCNA (Fig. 8B), yielding predicted docking scores of -270.56, -238.06, and -253.56, respectively. A more negative docking score indicates a higher probability of binding between the two proteins. Additionally, a positive correlation between RAD18 and Polη was observed using the CorrelationAnalyzeR tool (Fig. 8C). RAD18, Polη, and ubiquitinated PCNA (ub-PCNA) were detected in co-immunoprecipitates obtained from lysates using a Polη-specific antibody, indicating a direct interaction between the three proteins (Fig. 8D). Given that RAD18-mediated monoubiquitination of PCNA likely facilitates the recruitment of Polη to DNA lesions, we expected reduced Polη recruitment in RAD18 knockdown cells. To test this hypothesis, we treated control and RAD18 knockdown OVCAR3 cells with cisplatin, followed by immunofluorescence staining. Confocal image analysis revealed that cisplatin-treated control cells showed increased colocalization of RAD18 and Polη in the nucleus of OVCAR3 cells compared to RAD18 knockdown cells (Fig. 8E). Overall, this finding suggests that RAD18 positively regulates the recruitment of Polη to DNA damage sites and therefore, targeting RAD18 with miR-379-5p could inhibit the TLS pathway in ovarian CSCs.

## Discussion

Acquired chemoresistance poses a significant challenge in cancer treatment, especially in OC as it leads to frequent tumor relapse and higher mortality rates [22, 25]. Thus, improving therapeutic outcomes requires strategies that target chemoresistance, specifically CSCs. MicroRNAs play a vital role in the maintenance of CSCs and are a critical regulator of the progression of cancer [7]. miRNAs have the ability to regulate CSCs by either promoting or inhibiting their activity [26]. Recently, researchers have shown increasing interest in miR-379-5p due to its tumor-suppressor ability in various cancer types [27]. The downregulation of miR-379-5p has been directly linked to poor patient outcomes in multiple cancers [27, 28]. miR-379-5p exerts its tumor-suppressive function by inhibiting cell proliferation, blocking epithelial-to-mesenchymal transition (EMT), and preventing metastasis and invasion. However, its specific role in regulating CSCs remains largely unexplored.

Our study provides evidence that overexpression of miR-379-5p inhibits the growth of ovarian CSCs, suggesting its potential role in tumor maintenance and progression. It is worth noting that cisplatin treatment often enriches the CSC subpopulation [29]. Overexpression of miR-379-5p has been shown to significantly reduce the population of CSCs, as evidenced by the enrichment analysis of CD44 + CD117+ cells. Additionally, miR-379-5p overexpression enhances the effectiveness of cisplatin treatment by sensitizing ovarian CSCs. While previous studies have reported the inhibition of DNA repair by miR-379-5p through targeting PARP1, XRCC6 [30], and KIF4 [31], our study reveals, for the first time, that miR-379-5p can inhibit DNA repair by blocking the TLS pathway.

Each miRNA can regulate multiple targets, allowing it to exert a wide range of effects. In our study, we identified RAD18 as a critical target of miR-379-5p, underscoring its role in inhibiting chemoresistance in ovarian CSCs. RAD18, an E3 ubiquitin ligase, is integral to the error-prone TLS repair mechanism, which can promote carcinogenesis and tumor progression [32, 33]. Elevated RAD18 expression has been linked to reduced 5-year survival rates in cancer patients [33, 34]. RAD18 has also been associated with enhanced EMT in colorectal cancer [16] and contributes to mutagenesis [35]. In our study, we observed higher expression of RAD18 in spheroids derived from OC cells and HGSOC patient samples. Moreover, single-cell RNA analysis confirmed increased RAD18 expression in various ovarian cell types, suggesting its relevance to OC. Furthermore, the downregulation of RAD18 reduced the enrichment of CSCs and sensitized them to cisplatin treatment.

Our findings indicate that targeting RAD18 by miR-379-5p causes increased DNA damage accumulation. Additionally, we have shown that miR-379-5p overexpression has an inhibitory effect on the ability of ovarian CSCs to repair DNA. This reduced DNA repairability of CSCs is due to the inhibition of RAD18 activity by miR-379-5p, which prevents the recruitment of Polη to the DNA lesion site, thereby inhibiting the lesion bypass by TLS polymerases. It is widely recognized that RAD18 promotes the recruitment of Polη at the damage site via monoubiquitinating-PCNA [36, 37]. Our study aligns with previous findings on RAD18-mediated Polη recruitment, specifically for CSCs. The targeting of RAD18 by miR-379-5p, therefore, disrupts the TLS pathway. In the absence of TLS repair, the persisting DNA lesions activate DNA damage response genes, which leads to cell cycle inhibition and apoptosis activation in CSCs.

## Conclusion

In summary, our study reveals that the downregulation of miR-379-5p in CSCs promotes the recruitment of RAD18-mediated Pol η to DNA lesion sites, thereby initiating the TLS pathway. TLS pathway is a significant contributor to decreased platinum drug sensitivity and chemoresistance, which often leads to tumor relapse in OC patients. Restoring miR-379-5p expression in CSCs represents a potential strategy to eliminate CSCs, prevent ovarian metastasis, and reduce the risk of tumor recurrence. This research highlights a novel molecular mechanism that could be targeted to overcome chemoresistance in OC.

## Supplementary information

Full Blots

Supplementary material

## Data Availability

The datasets generated during or analyzed during the current study are available from the corresponding author upon reasonable request.
