# Supplementary material for: MicroRNA-379-5p attenuates cancer stem cells and reduces cisplatin resistance in ovarian cancer by regulating RAD18/Polη axis
Source: Cell Death Dis. 2025 Feb 27;16(1):140. doi: 10.1038/s41419-025-07430-5 (PMC11868536; doi:10.1038/s41419-025-07430-5)

Fig. 4G

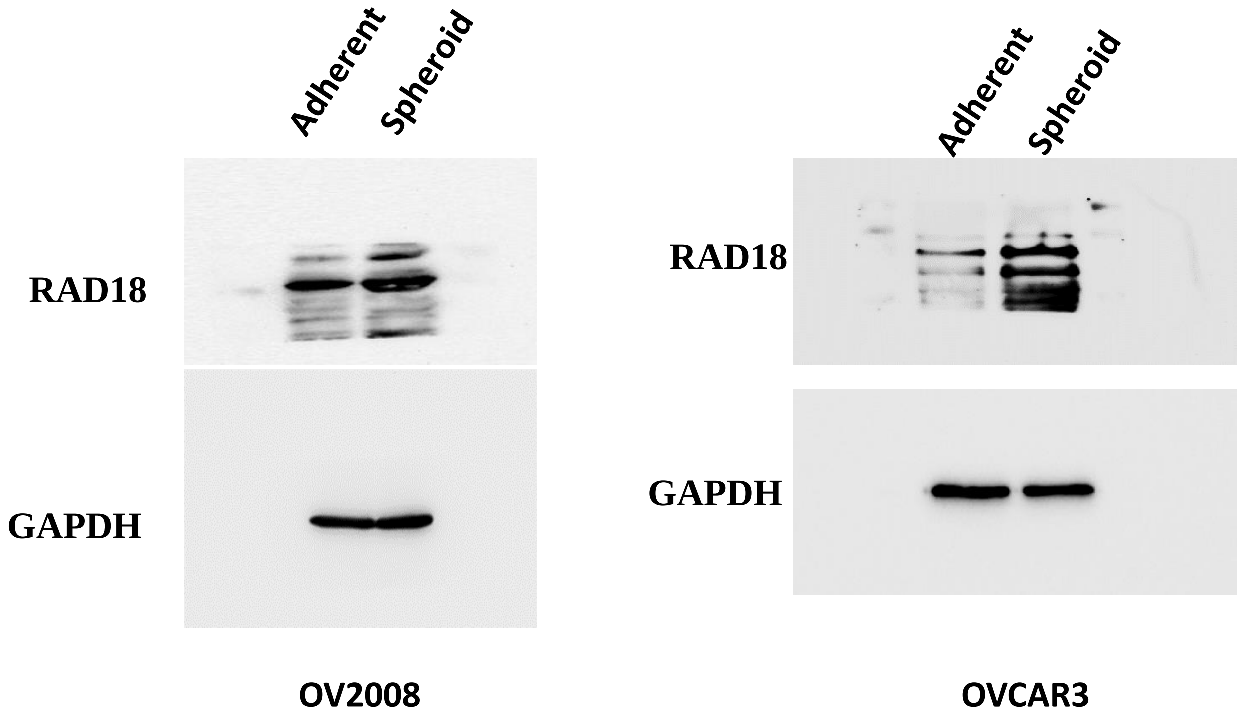
Adherent
Spheroid
RAD18
GAPDH
OV2008
Adherent
Spheroid
RAD18
GAPDH
OVCAR3

Fig. 5C

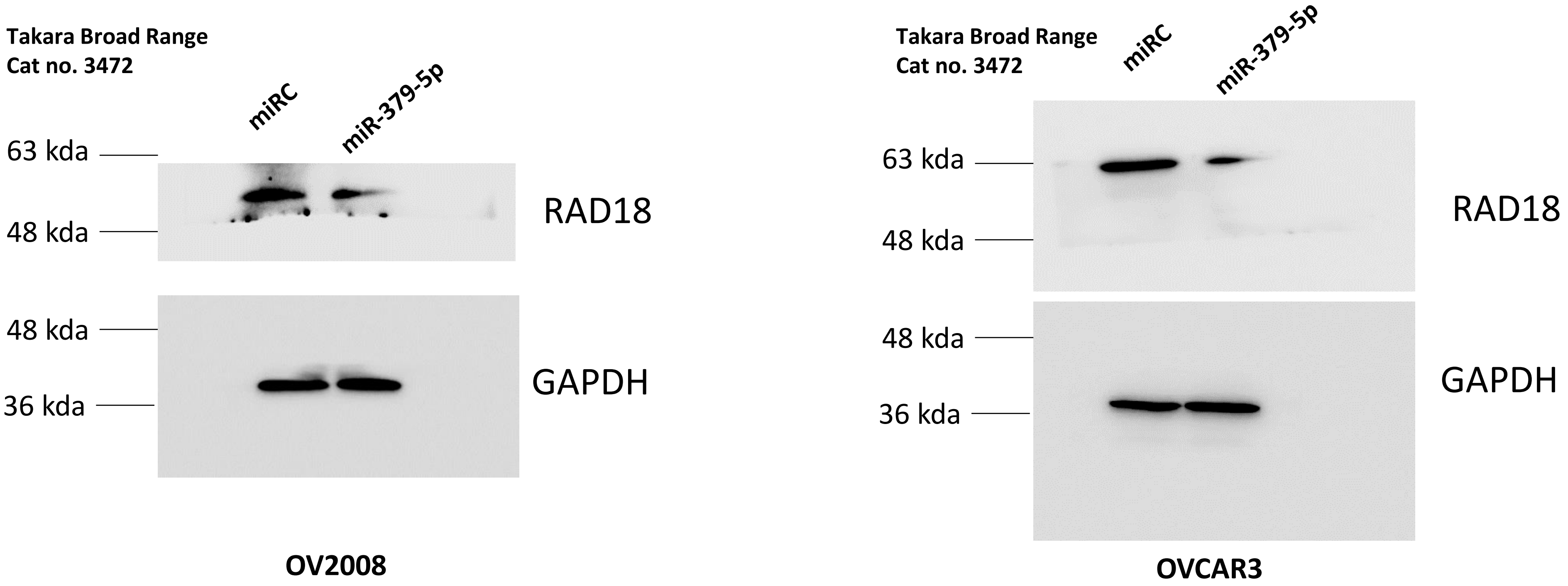
Takara Broad Range
Cat no. 3472
miRC
miR-379-5p
63 kda
48 kda
RAD18
48 kda
36 kda
GAPDH
OV2008
Takara Broad Range
Cat no. 3472
miRC
miR-379-5p
63 kda
48 kda
RAD18
48 kda
36 kda
GAPDH
OVCAR3

Fig. 7C

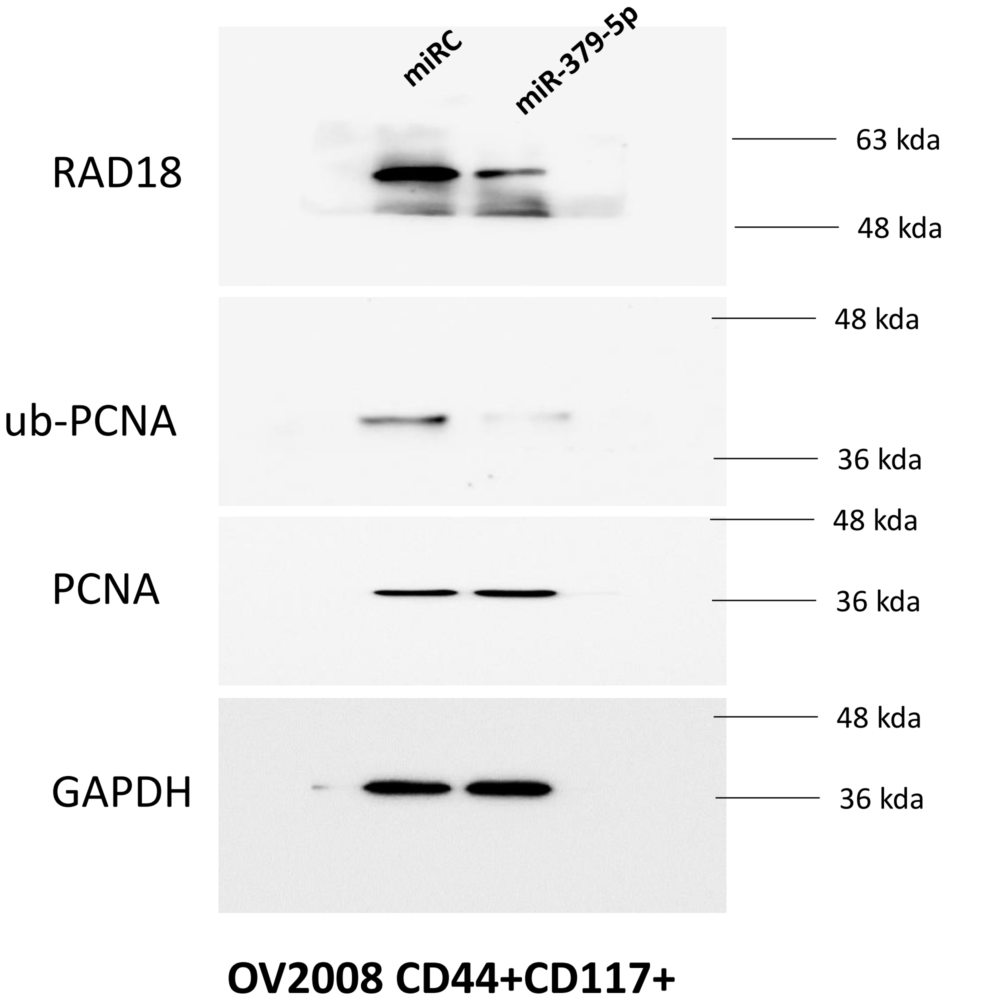
Takara Broad Range
Cat no. 3472
miRC
miR-379-5p
RAD18
63 kda
48 kda
ub-PCNA
48 kda
36 kda
PCNA
48 kda
36 kda
GAPDH
48 kda
36 kda
OV2008 CD44+CD117+

Fig. 7E

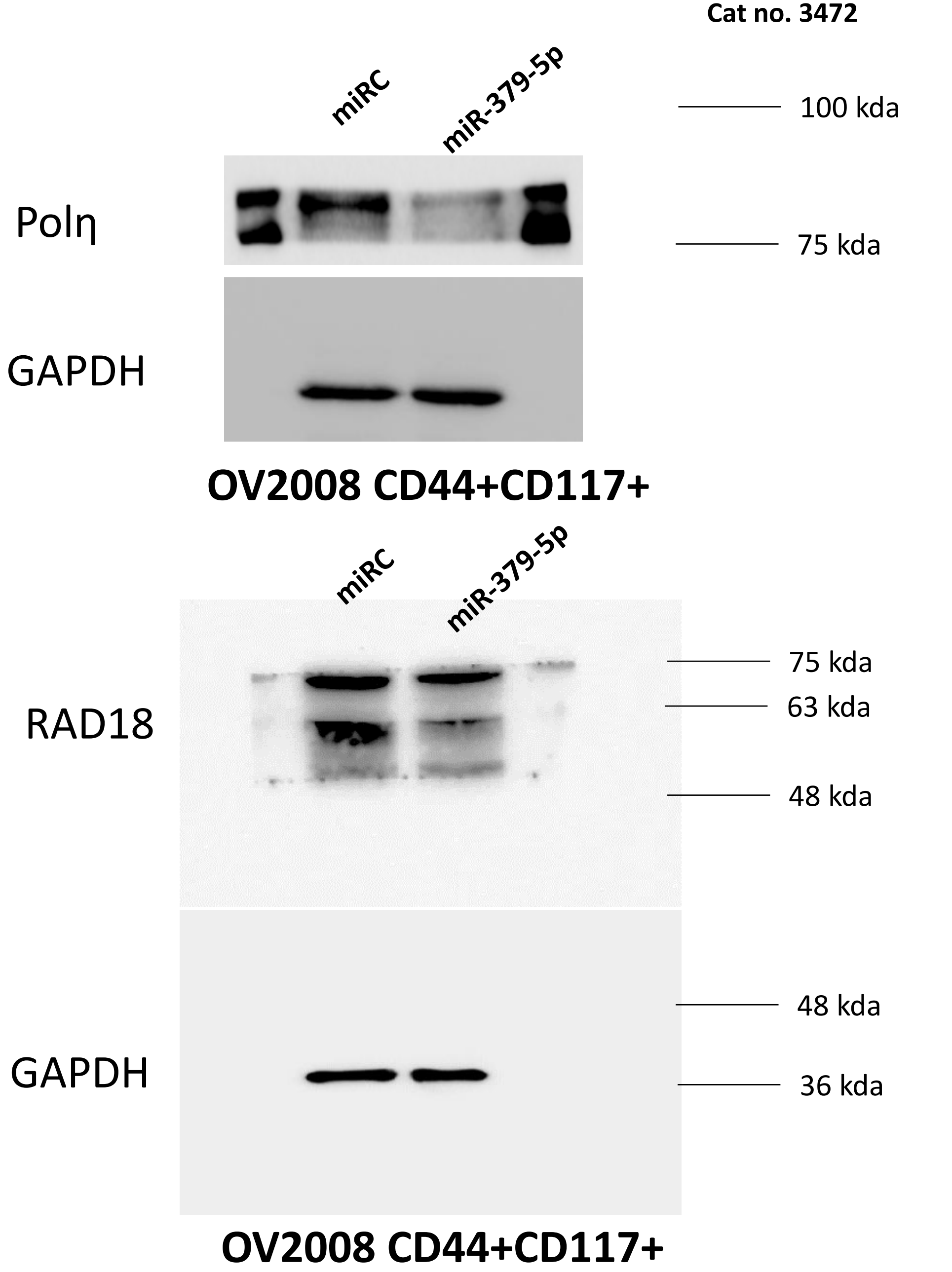
Takara Broad Range
Cat no. 3472
miRC
miR-379-5p
100 kda
Polη
75 kda
GAPDH
OV2008 CD44+CD117+
miRC
miR-379-5p
RAD18
75 kda
63 kda
48 kda
GAPDH
48 kda
36 kda
OV2008 CD44+CD117+

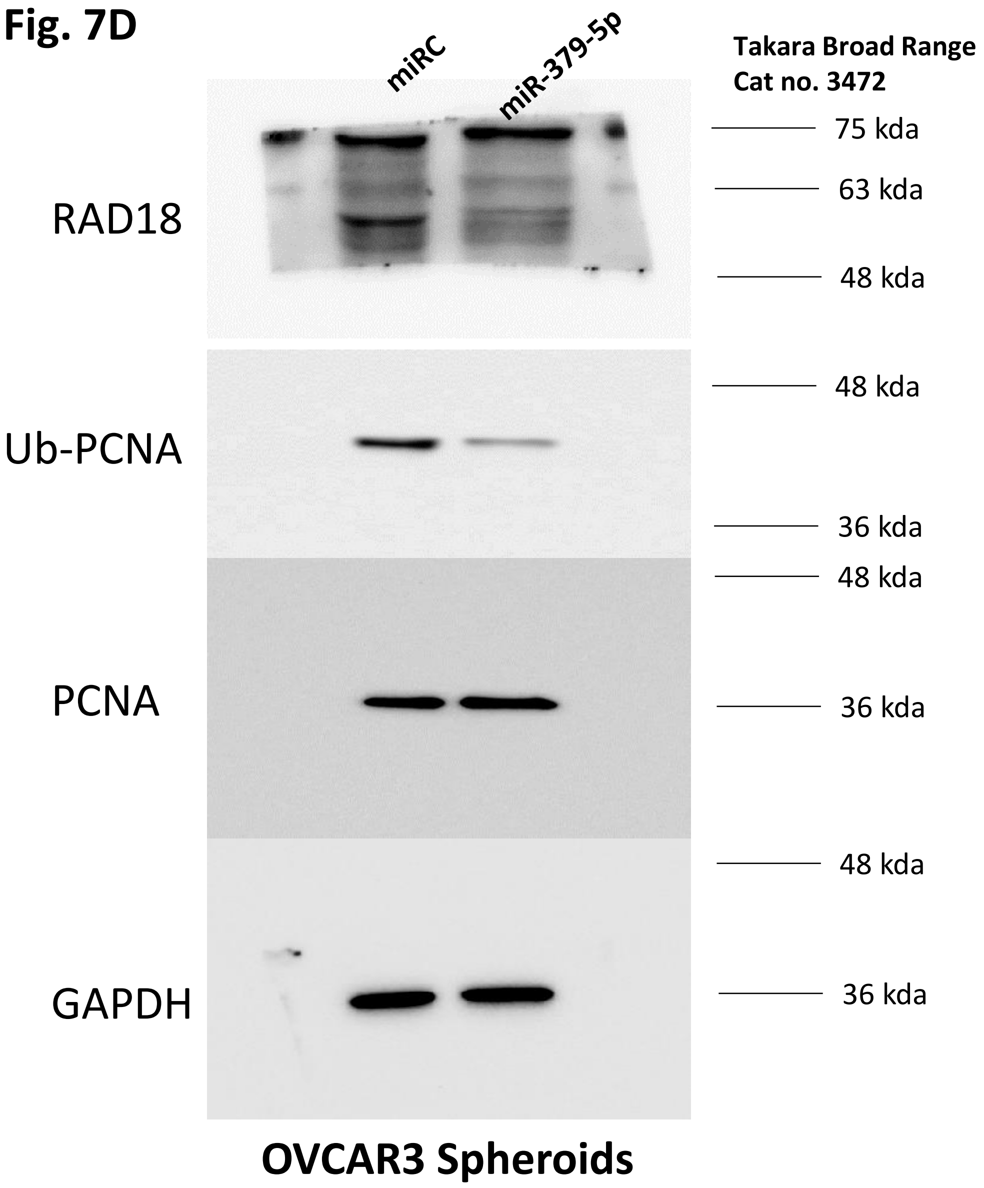
Fig. 7D
miRC
miR-379-5p
Takara Broad Range
Cat no. 3472
RAD18
75 kda
63 kda
48 kda
Ub-PCNA
48 kda
36 kda
PCNA
48 kda
36 kda
GAPDH
48 kda
36 kda
OVCAR3 Spheroids

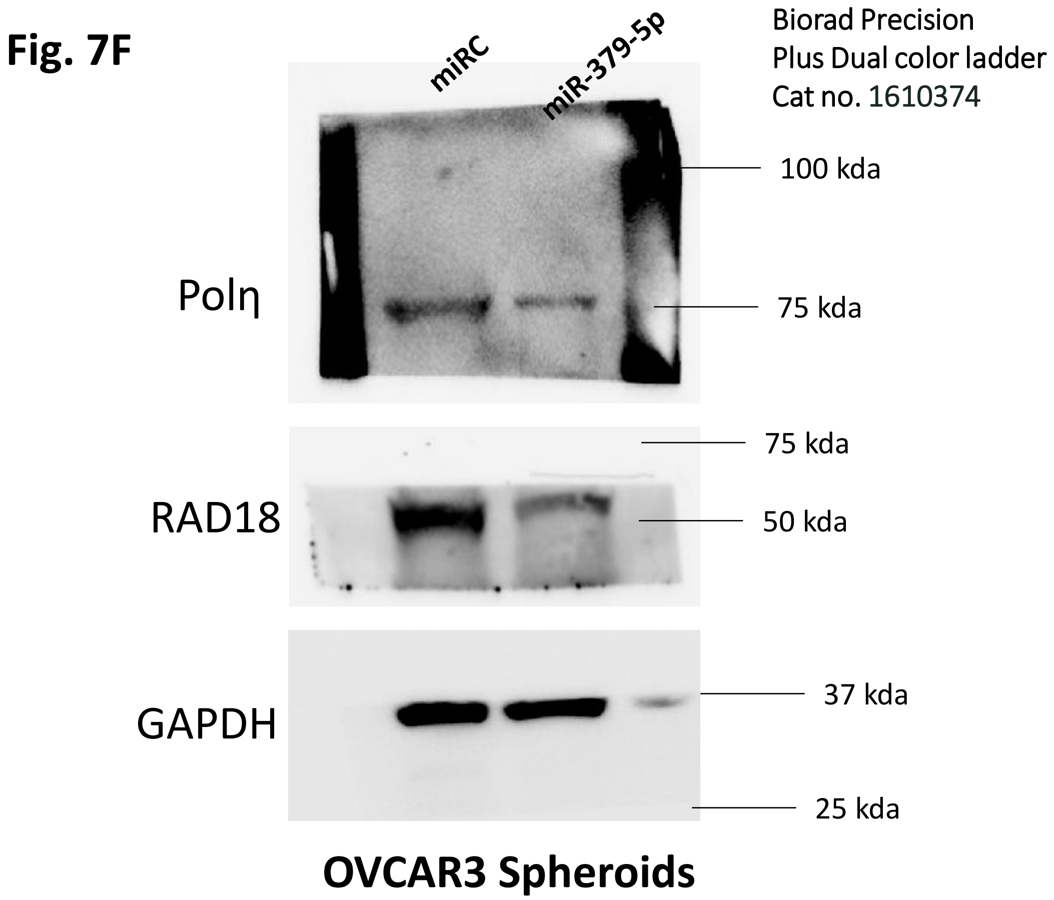
Fig. 7F
miRC
miR-379-5p
Biorad Precision
Plus Dual color ladder
Cat no. 1610374
Polη
100 kda
75 kda
RAD18
75 kda
50 kda
GAPDH
37 kda
25 kda
OVCAR3 Spheroids

Fig. 7G

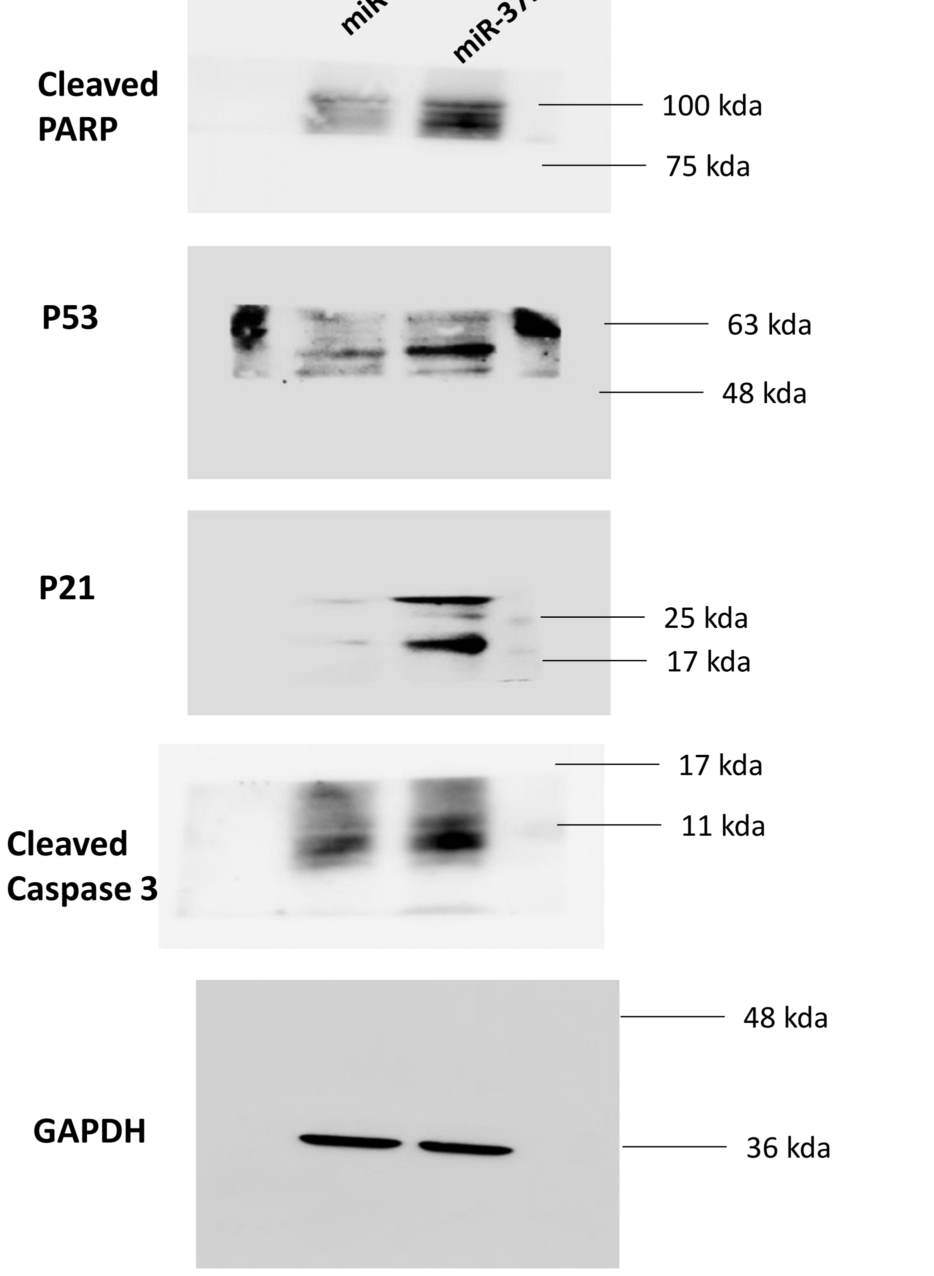

OV2008 CD44+CD117+

Fig. 7H

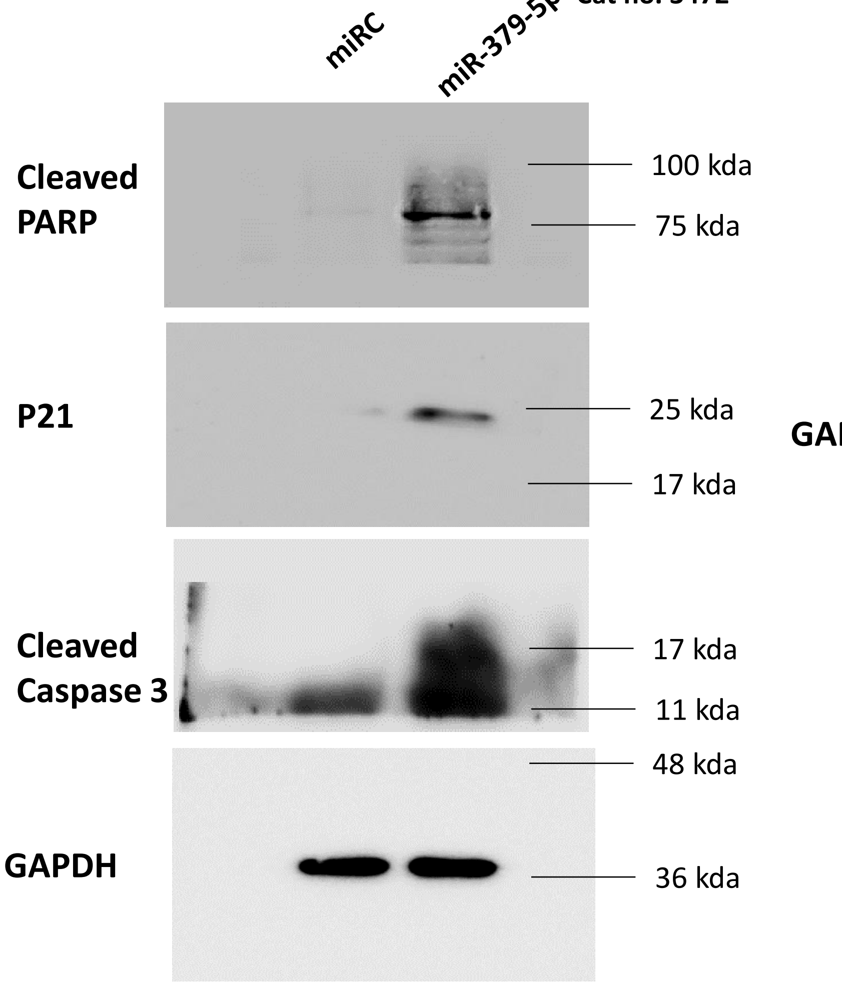

OVCAR3 Spheroids

Fig. 7I

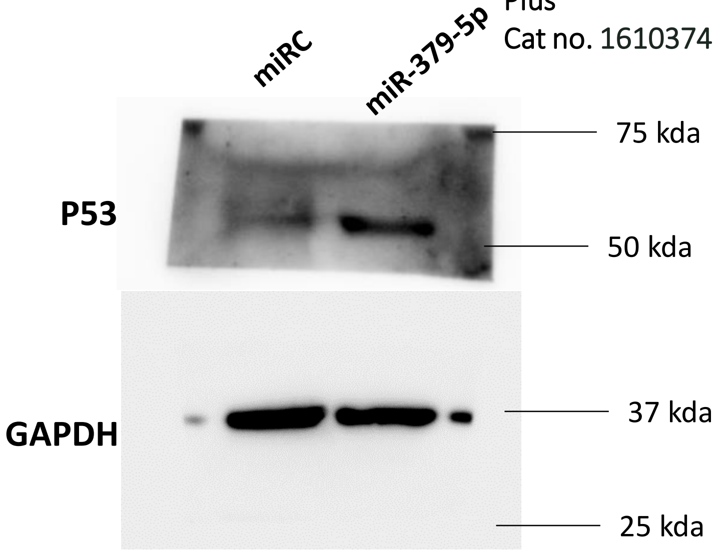

OVCAR3 Spheroids

Fig. 7K

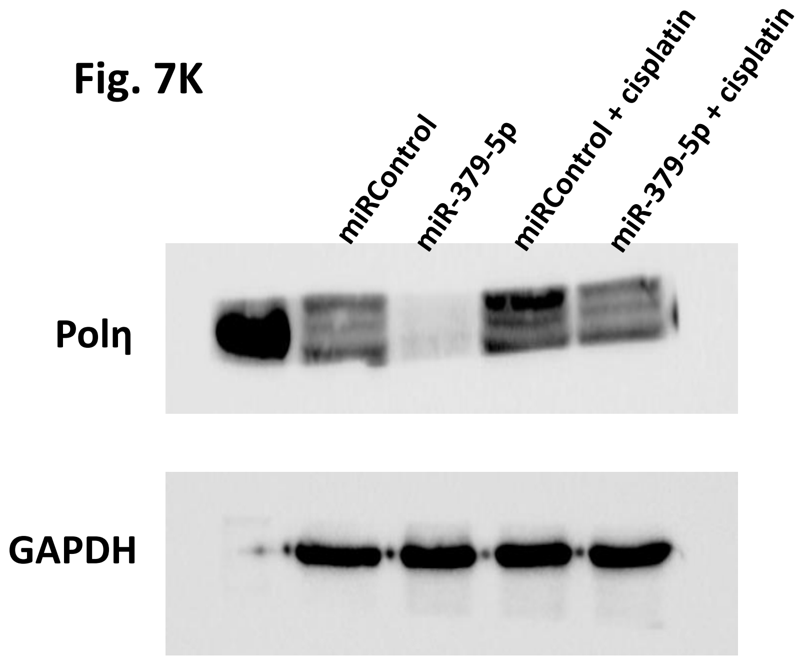

Nuclear Fraction

Fig. 7L

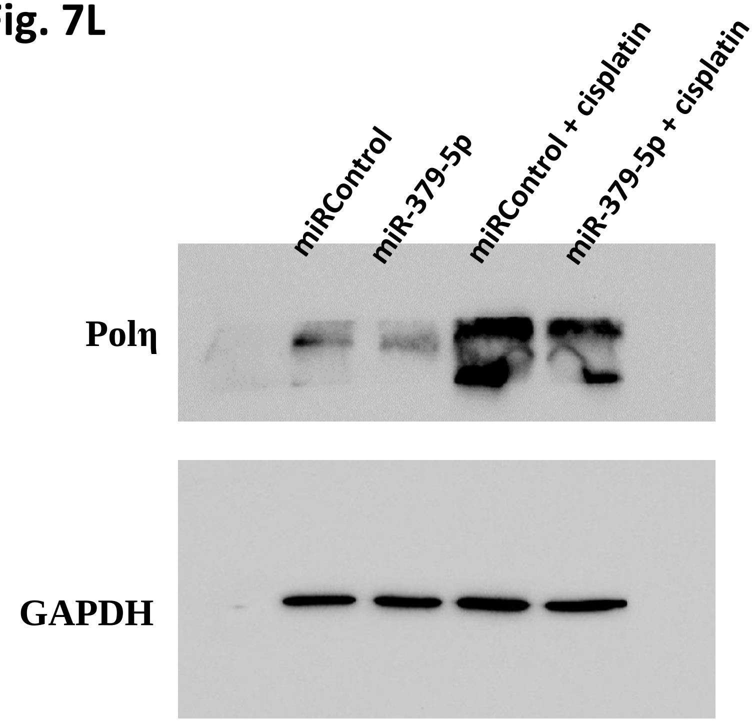

Whole Cell Lysate

Fig. 8D

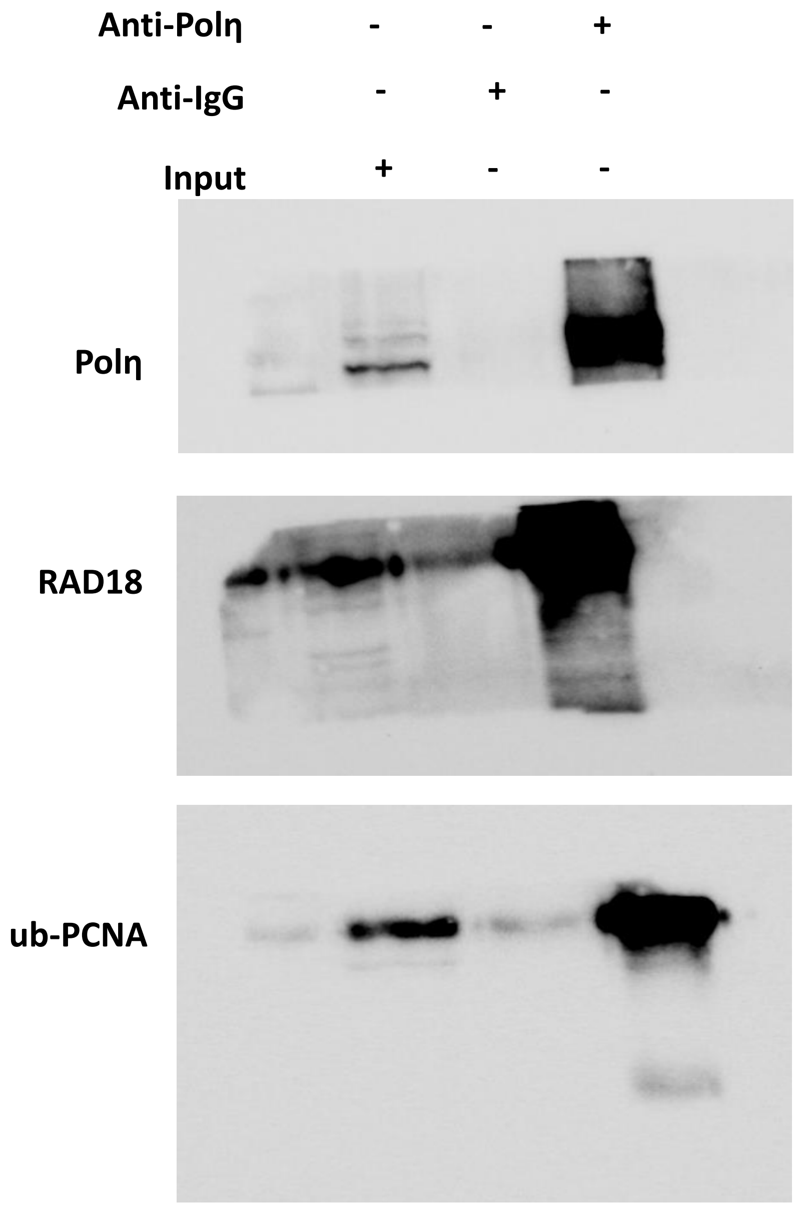

Supplement: Supplementary file 1 — Full Blots [file 41419_2025_7430_MOESM1_ESM.pdf]
