# Supplementary material for: MicroRNA-379-5p attenuates cancer stem cells and reduces cisplatin resistance in ovarian cancer by regulating RAD18/Polη axis
Source: Cell Death Dis. 2025 Feb 27;16(1):140. doi: 10.1038/s41419-025-07430-5 (PMC11868536; doi:10.1038/s41419-025-07430-5)
Supplement: Supplementary file 2 — Supplementary material [file 41419_2025_7430_MOESM2_ESM.pdf]

MicroRNA-379-5p Attenuates Cancer Stem Cells and Reduces Cisplatin Resistance in Ovarian Cancer by Regulating RAD18/Polη Axis

Devendra Shukla[1,2*], Sanjay Mishra[3*], Tanima Mandal[1,2], Manish Charan[3], Ajeet Kumar Verma[3], Md Maqsood Ahamad Khan[4], Nabanita Chatterjee[5], Amit Kumar Dixit[6], Senthil Kumar Ganesan[2,4], Ramesh K. Ganju[3#], Amit Kumar Srivastava[1,2#].

* Joint First Authorship

#Correspondence: Amit Kumar Srivastava, Cancer Biology and Inflammatory Diseases, CSIR-Indian Institute of Chemical Biology, Kolkata,-700032, WB, India. Phone +917651879029, e-mail: amit@iicb.res.in
Ramesh Ganju, Department of Pathology, The Ohio State University, USA. e-mail: Ramesh.Ganju@osumc.edu

1. Materials and methods:

1.1 Live dead imaging

Ovarian cancer spheroids were transfected with either miR-379-5p or siRAD18 and subsequently treated with cisplatin or PBS for 12 hours. After treatment, the cells were washed and resuspended in serum-free medium. They were then stained with CFDA (Invitrogen V12883) and PI and examined under a fluorescence microscope.

1.2. Colony Formation Assay:

OV2008 and OVCAR3 cells were transfected with either the miR-379-5p mimic or miRC and seeded at a density of 1,000 cells per well in a 6-well plate. The cells were cultured for 10 days, after which the medium was aspirated, and the cells were washed with 1X PBS and fixed in 4% PFA for 1 hour. Following fixation, the cells were stained with 0.5% methylene blue for 4 hours. Colonies were counted, and the colony formation efficiency was calculated.

1.3 Spheroid formation assay

OV2008 CD44+CD117+ cells and OVCAR3 spheroids were transfected with either miR-Control (miRC) or miR-379-5p mimic using Lipofectamine 2000. The transfected cells were

cultured in transfection medium for 12 hours, after which the medium was replaced with complete CSC-specific medium. The cells were then seeded in a 6-well ultra-low attachment dish at a density of 1,000 cells per well. After 7 days, spheroid formation was evaluated and counted using an Olympus CKX53 inverted microscope.

1.4 **Transwell migration assay**

OVCAR3 and OV2008 cells, resuspended in low-serum medium, were seeded at a density of 0.5×10^5 cells per insert in a Transwell plate (VWR, 89235-020). The lower wells were filled with high-FBS medium, and the cells were allowed to migrate for 24 hours. After the incubation period, the cells were fixed with 4% PFA and stained with 0.2% crystal violet. The inserts were examined under an Olympus microscope.

1.5. Wound healing assay

OVCAR3 and OV2008 cells, transfected with either the miR-379-5p mimic or miRC, were seeded at a density of 0.5×10^6 cells per well and cultured for 24 hours. A scratch was created in each well using a 100 μL pipette tip, and the cells were allowed to heal for 12 hours. Images were captured at 0 hour and 12 hours to evaluate the wound-healing ability of the cells.

1.6 **Single-cell data analysis**

Data retrieval:

scRNA-seq datasets were retrieved from publicly available Gene Expression Omnibus Database, NCBI. The analysis of GSE173682 (4 samples) datasets consists of 10x genomics based single cell landscape of human gynecologic malignancies and sequencing were carried out by Regner et al., 2021 using Illumina NextSeq 500 platform. (Table 1). We re-analyzed the data using various in silico tools. Seurat package" (version4.3.0) were utilized in "R" (version 4.2.1). Feature, barcode and matrix files of datasets were filtered in a certain parameter [>200

genes and < 8000 genes; >400 unique Molecular Identifier (UMIs) and <10% mitochondrial RNA (mtRNA)]. "LogNormalize" Function were used to normalized the matrix files and obtain normalized counts. "FindAllVariable", "ScaleData" and "RunPCA" function "FindAllMarkers" were utilized to determine the high variable features, linear transformation, dimentionality reduction, principal component analysis and differentially expressed genes (DEGs). To reduce the dimension, top 20 principal component were considered in t-Distributed Stochastic Neighbor Embedding (t-SNE) technique. Louvain Modularity Optimization Algorithm were performed at a resolution of 0.5 to detect the cluster of cells (Blondel et al, 2008). cell types annotation was performed manually on the basis of cell markers and Human Proteome Atlas (Thul et al, 2018).

1.7. Bioinformatics analysis

Pan-cancer analysis of miR-379-5p expression was performed using CanceMIRNome (1). DIANA TOOLS miRPath (2), miRWalk (3) and miRmap (4) were used to identify a number of common targets of miR-379-5p. The miRNA-RAD18 binding duplex was obtained from RNAhybrid platform (5). Further, the RAD18 expression in different types of cancer was obtained from Gene Expression Profiling Interactive Analysis (GEPIA) tool (6) and UALCAN (7,8) . The correlation between RAD18 expression levels and patient survival in ovarian cancer was established from OncoLnc (9). The interactome of RAD18 was obtained from the STRING- Database (10).

Supplementary Tables

Table S1: List of reagents used in this study.

Reagents	Catalogue No.	Company
RPMI	11875-085	Gibco
Pen/Strep	10378-016	Gibco
FBS	10082-147	Gibco
DMEM/F12 KO Media	10565-018	Gibco
Replacement Serum	10828-028	Gibco
EGF	PHG0311	Gibco
bFGF	PHG0264	Gibco
MycoAlert Kit	LT07-318	Lonza
Collagenase type IV	07427	Stem Cell Technologies
psiCHECK2 vector	C8021	Promega
Lipofectamine 2000	11668019	Invitrogen
Propidium iodide	P4170	Sigma
PowerUp™ SYBR™ Green Master Mix	A25742	Applied Biosystems
TaqMan Universal PCR Master mix	4324018	Applied Biosystems
cDNA Reverse Transcription	4368814	Applied Biosystems
anti-Rabbit AlexaFluor488 antibody	AB150077	Abcam
Rabbit Specific HRP/DAB Detection kit	AB64261	Abcam
Protease inhibitor cocktail	635672	Takara
Protein A agarose beads	20333	Thermo Fisher
Dual-Luciferase Reporter Assay System	E1910	Promega

Table S2: List of primers used for qPCR

Genes	Forward	Reverse
Oct4	5'- AACAGGGAATGGGTGAATGA-3'	5'- TAGAAGTGCCTCTGCCTTCC-3'
Sox2	5'-TTGCTGCCTCTTTAAGACTAGGA-3'	5'- TAAGCCTGGGGCTCAAACT-3'
Nanog	5′-GTCCCAAAGGCAAACAACCC-3′	5′-TTGACCGGGACCTTGTCTTC-3′
Rad18	5'- CAGCTGTTTATCACGCGAAG-3'	5'-TTAAATCACGATCAGAGAGCAAA-3'
18S	5'-GCAATTATTCCCCATGAACG-3'	5'-TGTACAAAGGGCAGGGACTTA-3'

Table S3: List of primers used for dual luciferase assay.

Genes	Forward
Rad18 3' UTR WT FP	5'- TT GCGGCCGC GCC ATG GGTATGGACCTTAAAGGGGGCATTTCTCC -3'
Rad18 3' UTR WT RP	5'- CCC CTCGAG AGCTCCATCAGGGCAGGAACTGTC -3'
Rad18 3' UTR MUT FP	5′- AGGAGGGCAGATGCTGTGCGAAGTCTGTCGGGGGG-3'
Rad18 3' UTR MUT RP	5'- CCCCCCGACAGACTTCGCACAGCATCTGCCCTCCT-3'

Table S4: List of antibodies used in this study

Antibody	Catalogue No.	Company
Anti-Rad18	NB100-56523SS	Novus Biologicals
Anti-GAPDH	5174	Cell Signalling Technology
Anti-ub-PCNA	13439	Cell Signalling Technology
Anti-PCNA	13110	Cell Signalling Technology
Anti-Polη	ab236450	Abcam
Anti-P53	2527	Cell Signalling Technology
Anti-P21	2947	Cell Signalling Technology
Anti-Cleaved PARP	5625	Cell Signalling Technology
Anti-Cleaved Caspase-3	9664	Cell Signalling Technology
Anti-Ki67	ab15580	Abcam
Anti-Phospho-H2AX	20E3	Cell Signalling Technology
Anti-Rad18	D2B8	Cell Signalling Technology

Table S5: Patient demographic information

Parameters	Frequency	Percentage
Age		
≤18 years	1	2.6
19-40 years	12	30.8
41-60 years	17	43.6
>60 years	9	23.1
<18	11	28.2
18-25	18	46.2
>25	7	17.9
NA	3	7.7
CEA (ng/ml)		
<5	19	48.7
≥5	20	51.3
CA-125 (U/ml)		
≤35	5	12.8
35.1-499.9	18	46.2
500-999.9	8	20.5
≥1000	8	20.5
CA-19.9 (U/ml)		
<28	18	46.2
≥28	21	53.8
Ascites level		
High	12	30.8
Moderate	14	35.9
Low	13	33.3
FIGO Stage		
Stage I	13	33.3
Stage II	11	28.2
Stage III	10	25.6
Stage IV	5	12.8

Table S6: Summary of single cell ovarian datasets and differentially expressed genes (DEGs)

Cancer Type	Accession ID	Dataset	Platform	Sample	DEGs
Ovarian	GSE173682	Single cell (scRNA-seq)	Illumina	Non-metastatic- 02<br>Metastatic- 02	23627

Table S7: Predicted docking Score of protein-protein docking for Rad18-Polη, Rad18-PCNA and PCNA- Polη.

Rad18- Polη

Rank	1	2	3	4	5	6	7	8	9	10
Docking Score	-270.56	-261.72	-235.02	-233.10	-230.16	-221.87	-218.38	-218.33	-207.89	-207.73
Confidence Score	0.9177	0.9033	0.8456	0.8405	0.8325	0.8081	0.7970	0.7968	0.7609	0.7604
Ligand rmsd (Å)	76.22	75.98	43.04	38.11	45.15	74.87	73.99	84.09	65.17	72.61

Rad18-PCNA

Rank	1	2	3	4	5	6	7	8	9	10
Docking Score	-238.06	-227.75	-227.09	-226.38	-221.84	-217.24	-214.83	-210.35	-210.10	-206.92
Confidence Score	0.8534	0.8256	0.8237	0.8217	0.8080	0.7933	0.7853	0.7698	0.7689	0.7574
Ligand rmsd (Å)	97.05	46.06	104.42	106.52	73.61	80.12	80.56	78.22	99.95	66.25

PCNA- Polη

Rank	1	2	3	4	5	6	7	8	9	10
Docking Score	-253.86	-233.61	-229.64	-224.77	-213.10	-208.76	-208.59	-205.07	-203.93	-202.23
Confidence Score	0.8887	0.8419	0.8310	0.8169	0.7794	0.7641	0.7635	0.7505	0.7462	0.7397
Ligand rmsd (Å)	88.95	93.79	93.57	176.70	103.09	99.02	96.84	97.98	82.58	71.69

Supplementary Fig. 1

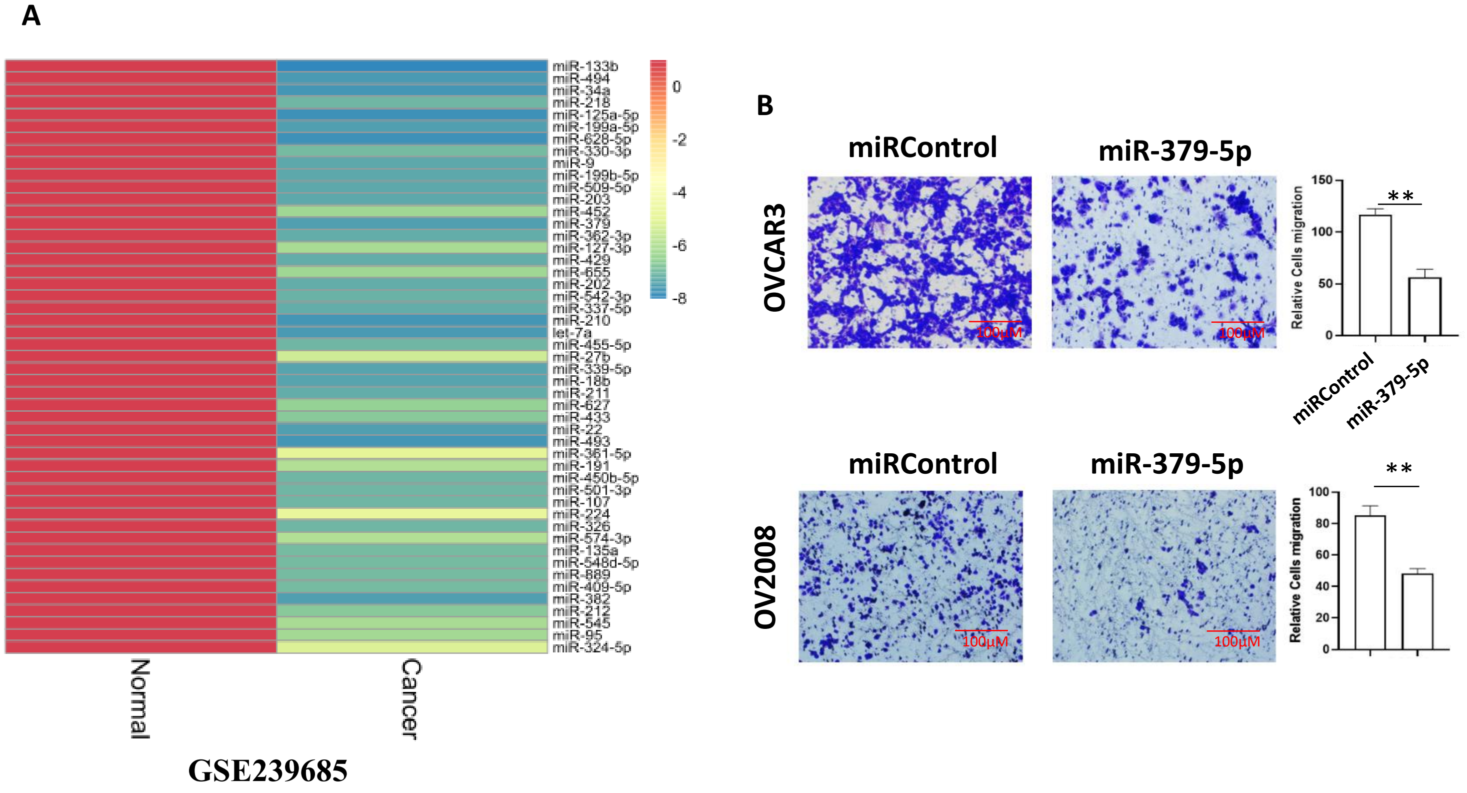

Fig. 1: (A) GEODataset GSE239685 shows the differentially expressing microRNAs in normal ovarian tissue samples vs ovarian cancer tissue. miR-379-5p is one of the most downregulated microRNAs in ovarian tumor samples. (B) Transwell migration assay on OVCAR3 and OV2008 monolayer bulk cells shows significantly higher migration in cells transfected with miRControl as compared to miR-379-5p overexpressing cells, indicating an anti-migration role played by miR-379-5p. The graphs were represented as mean ± S.D., n = 3, Significance levels $*p \leq 0.05$, $**p \leq 0.01$ and $***p \leq 0.001$.

Supplementary Fig. 2

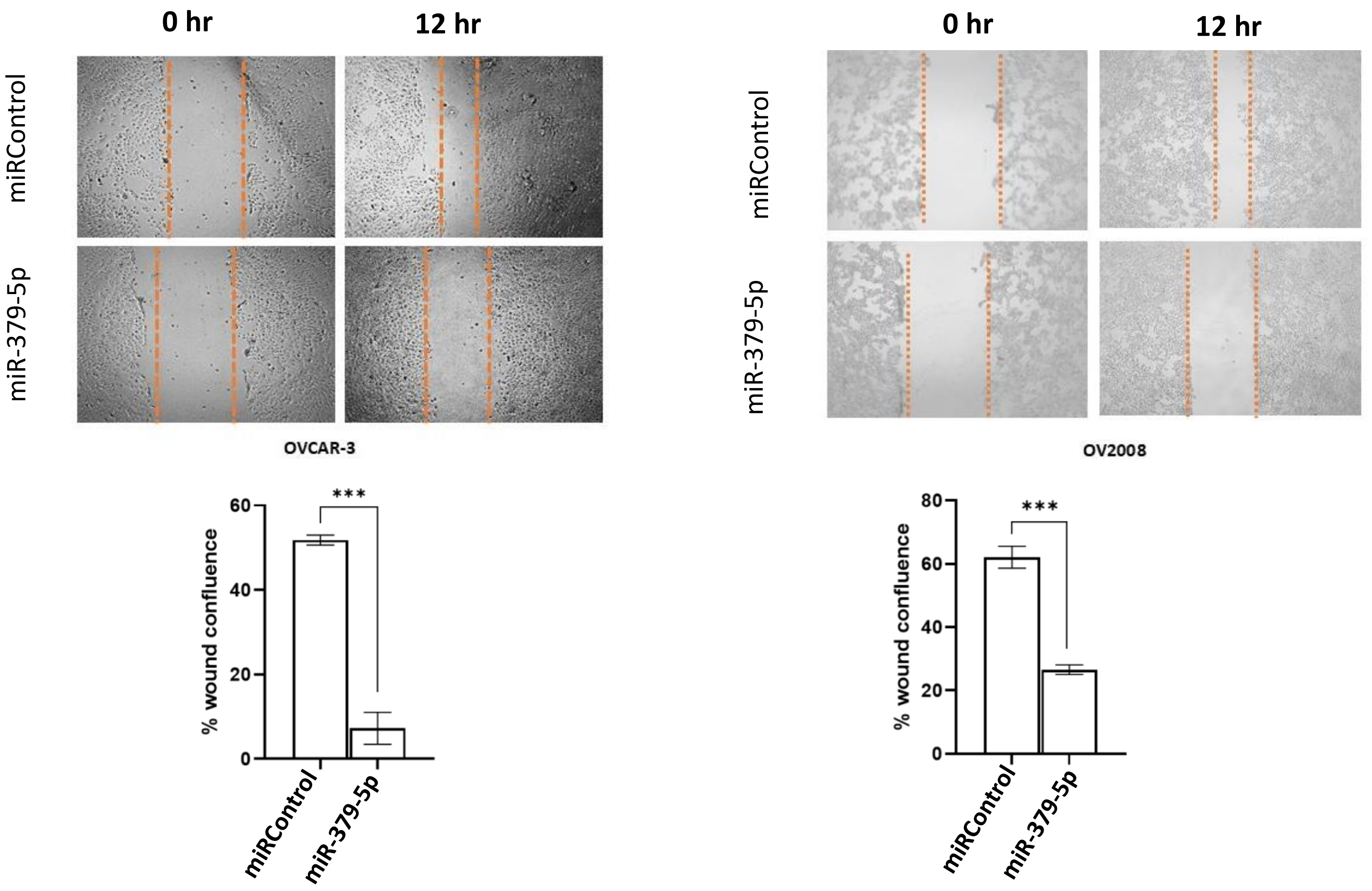

Fig. 2: Wound healing assay on OVCAR3 and OV2008 monolayer bulk cells showing significantly higher wound healing in cells transfected with miRControl cells compared to miR-379-5p overexpressing cells. The graphs were represented as mean ± S.D., n = 3, Significance levels $*p \leq 0.05$, $**p \leq 0.01$ and $***p \leq 0.001$.

Supplementary Fig. 3

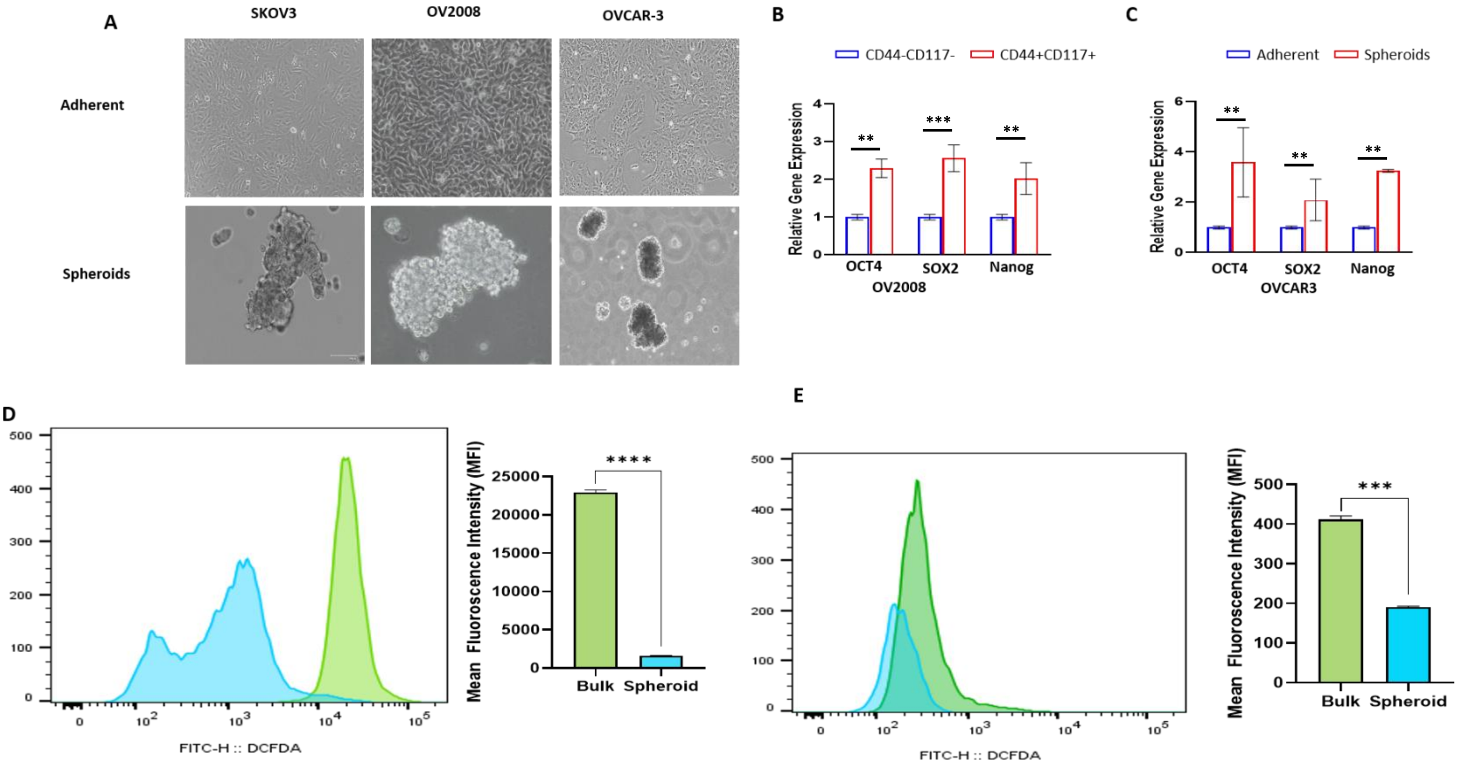

Fig. 3: (A) SKOV3, OV2008 and OVCAR3 cells were grown in adherent and spheroid cultures respectively; The stem cell marker genes expression analysis in (B) OV2008 and (C) OVCAR3 cells; Total ROS in (D) OVCAR3 adherent and spheroid, and (E) OV2008 adherent and spheroid, were measured via flow cytometry; The graphs were represented as mean ± S.D., n = 3, Significance levels *p≤ 0.05, **p≤ 0.01, ***p≤ 0.001 and ****p≤ 0.0001.

Supplementary Fig. 4

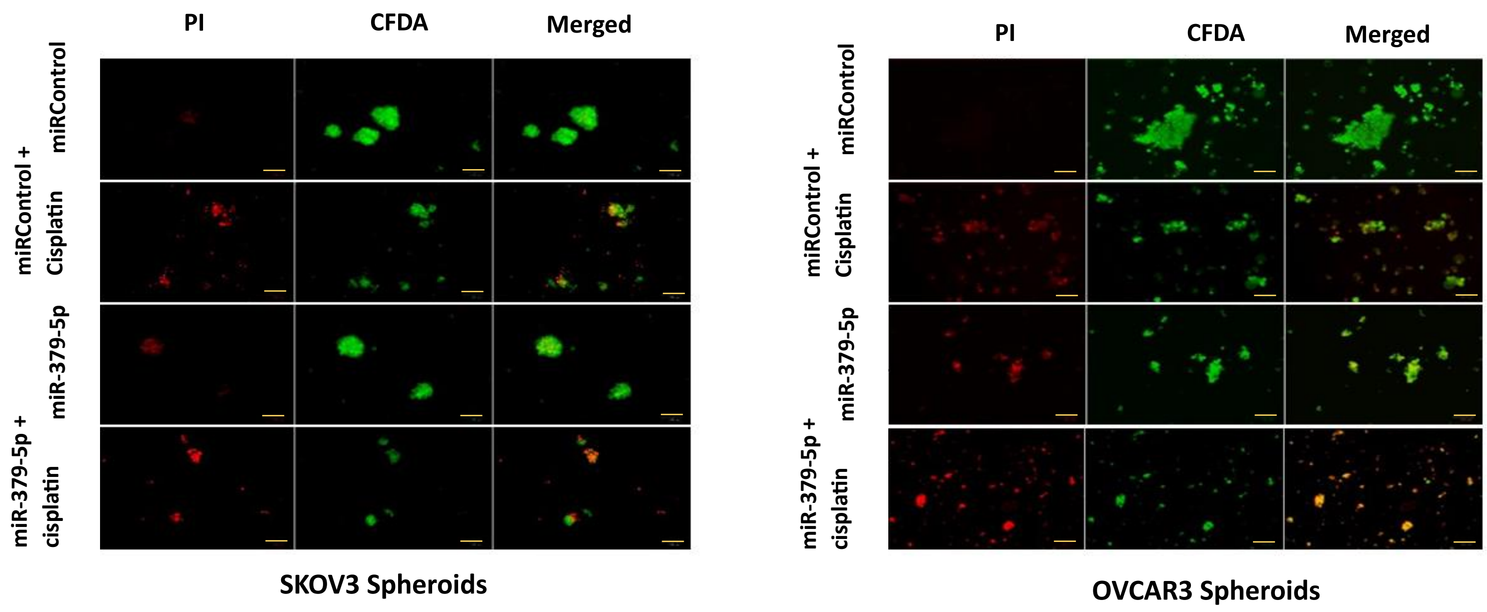

Fig. 4: PI/CFDA staining of SKOV3 and OVCAR3 cells showing live (green) or dead (red) cells. The miRControl and miR-379-5p transfected cells were treated with either PBS or 10μM cisplatin for 12 hours. The images were acquired using a fluorescence microscope. Scale bar = 100μM.

Supplementary Fig. 5

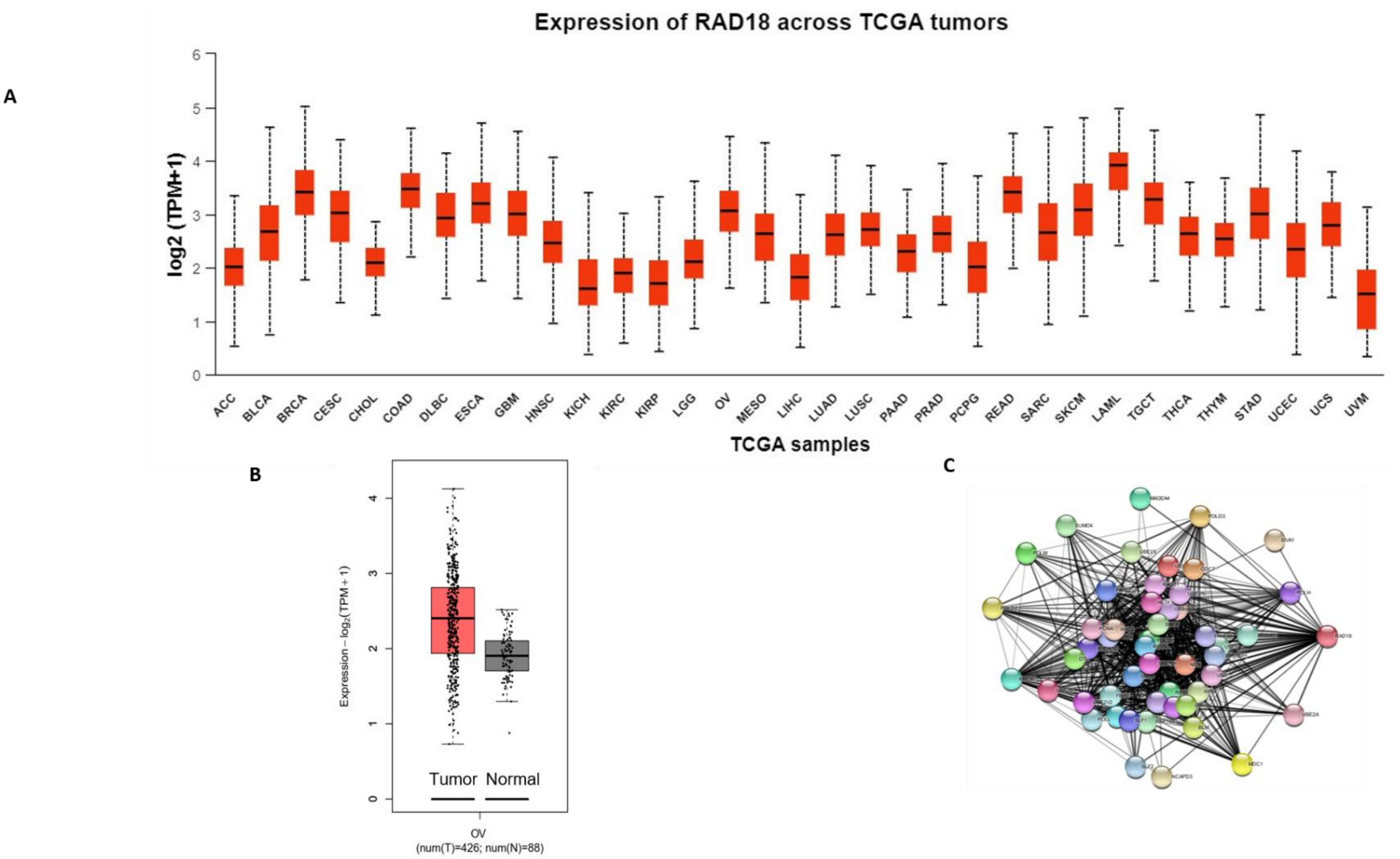

Fig. 5: (A) Pan cancer expression profiling of RAD18 gene samples retrieved from UALCAN; (B) Expression of RAD18 in tumor vs normal ovarian tissues reproduced from GEPIA. (C) TCGA survival curve analysis for RAD18 expression in ovarian cancer. (D) Protein-protein interaction network showing RAD18 interaction with POLH.

Supplementary Fig. 6

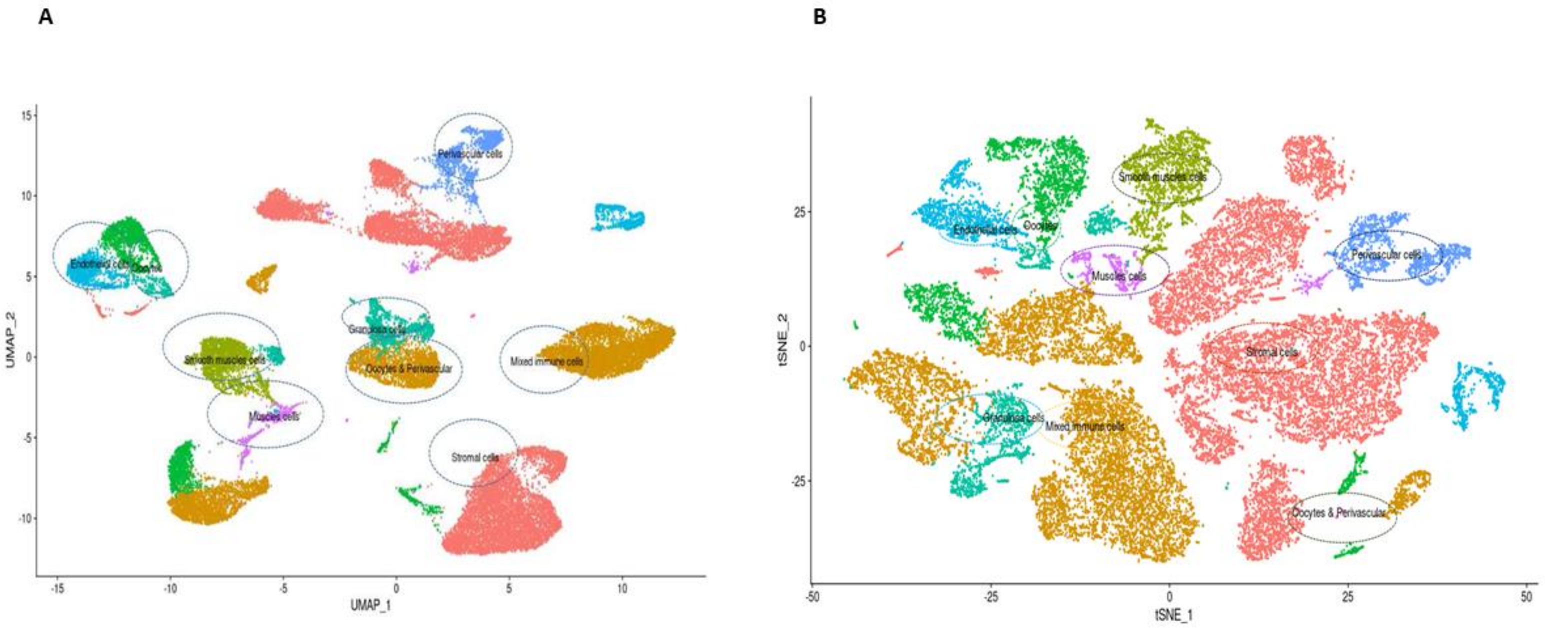

Fig. 6: (A) UMAP_2 plot showing the expression of RAD18 in different cell types; (B) tSNE_2 plot showing the expression of RAD18 among different cell types.

Supplementary Fig. 7

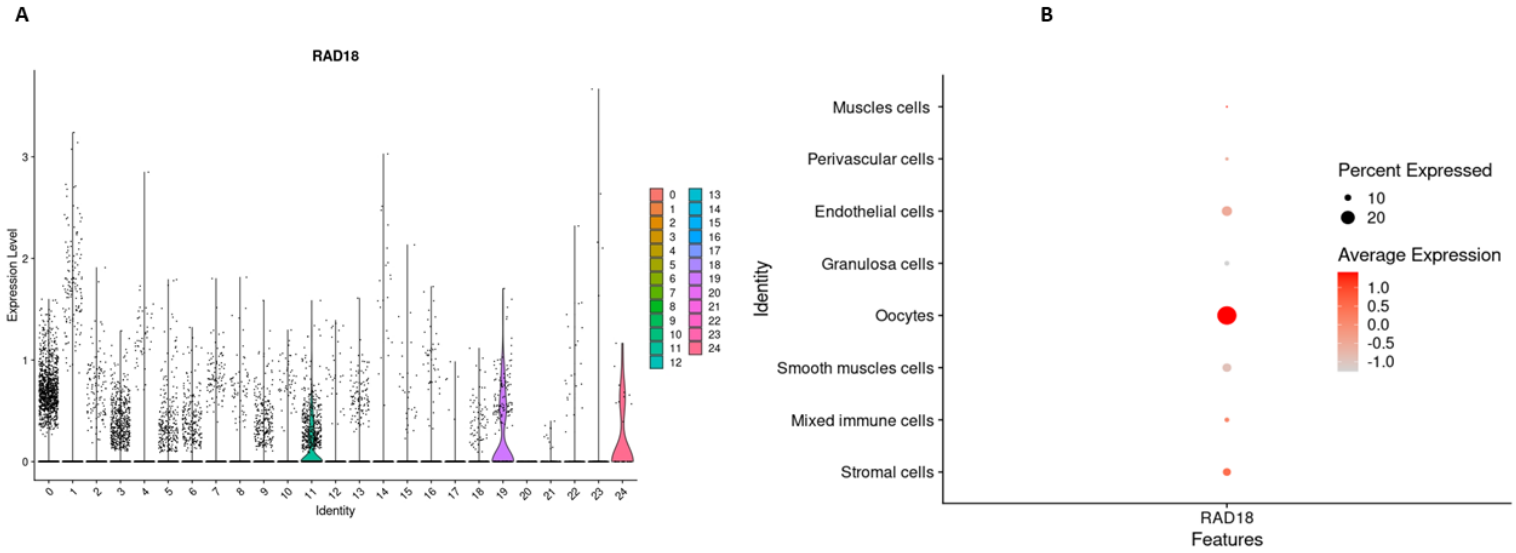

Fig. 7: (A) Violin plot showing the expression of RAD18 in metastatic vs non-metastatic condition in different cell types; (B) Dot plot showing the highest expression of RAD18 in metastatic condition in oocytes.

Supplementary Fig. 8

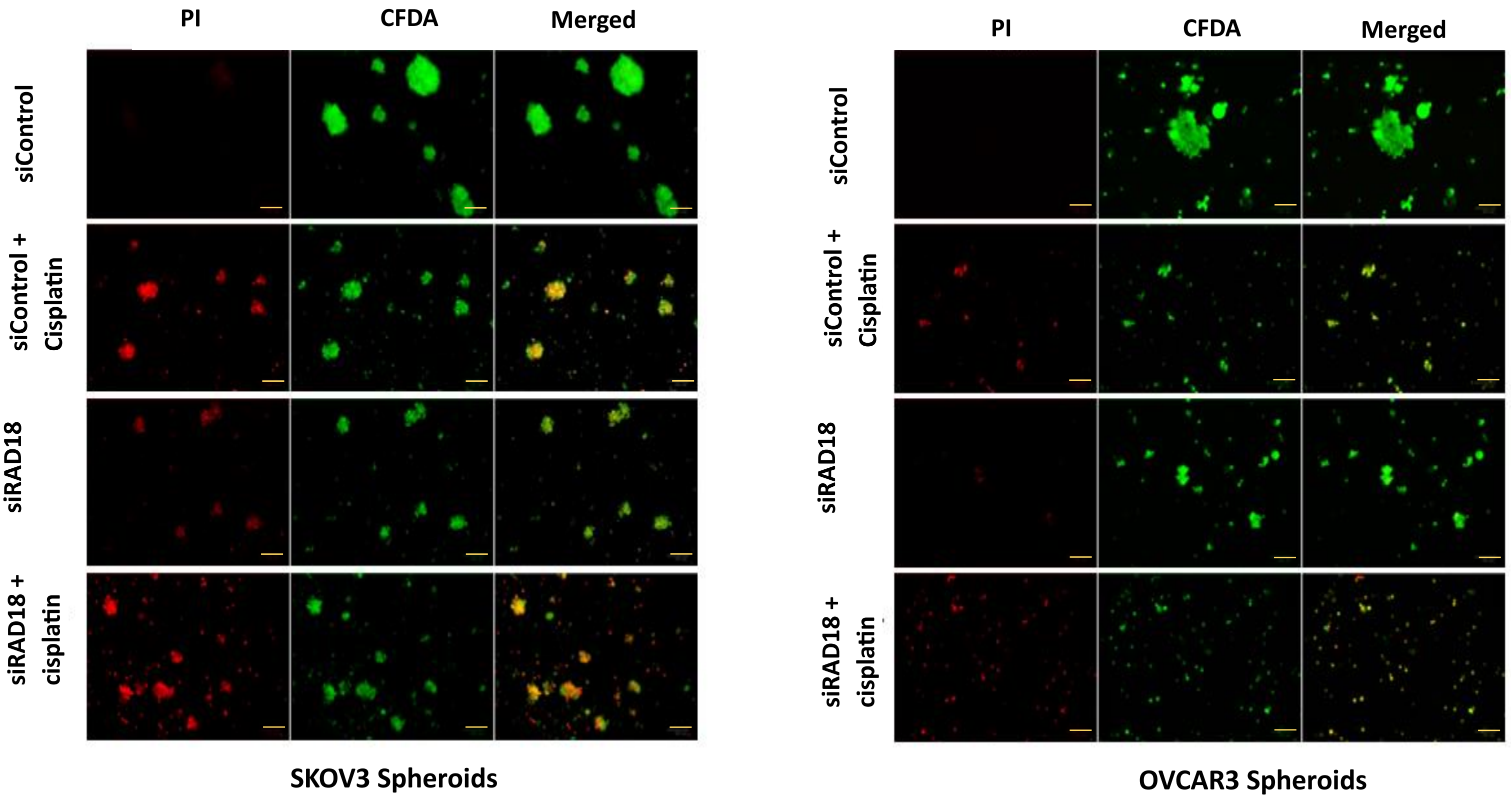

Fig. 8: PI/CFDA staining of SKOV3 and OVCAR3 cells showing live (green) or dead (red) cells. The siControl and siRAD18 transfected cells were treated with either PBS or 10µM cisplatin for 12 hours. The images were acquired using a fluorescence microscope. Scale bar = 100µM.

Supplementary Fig. 9

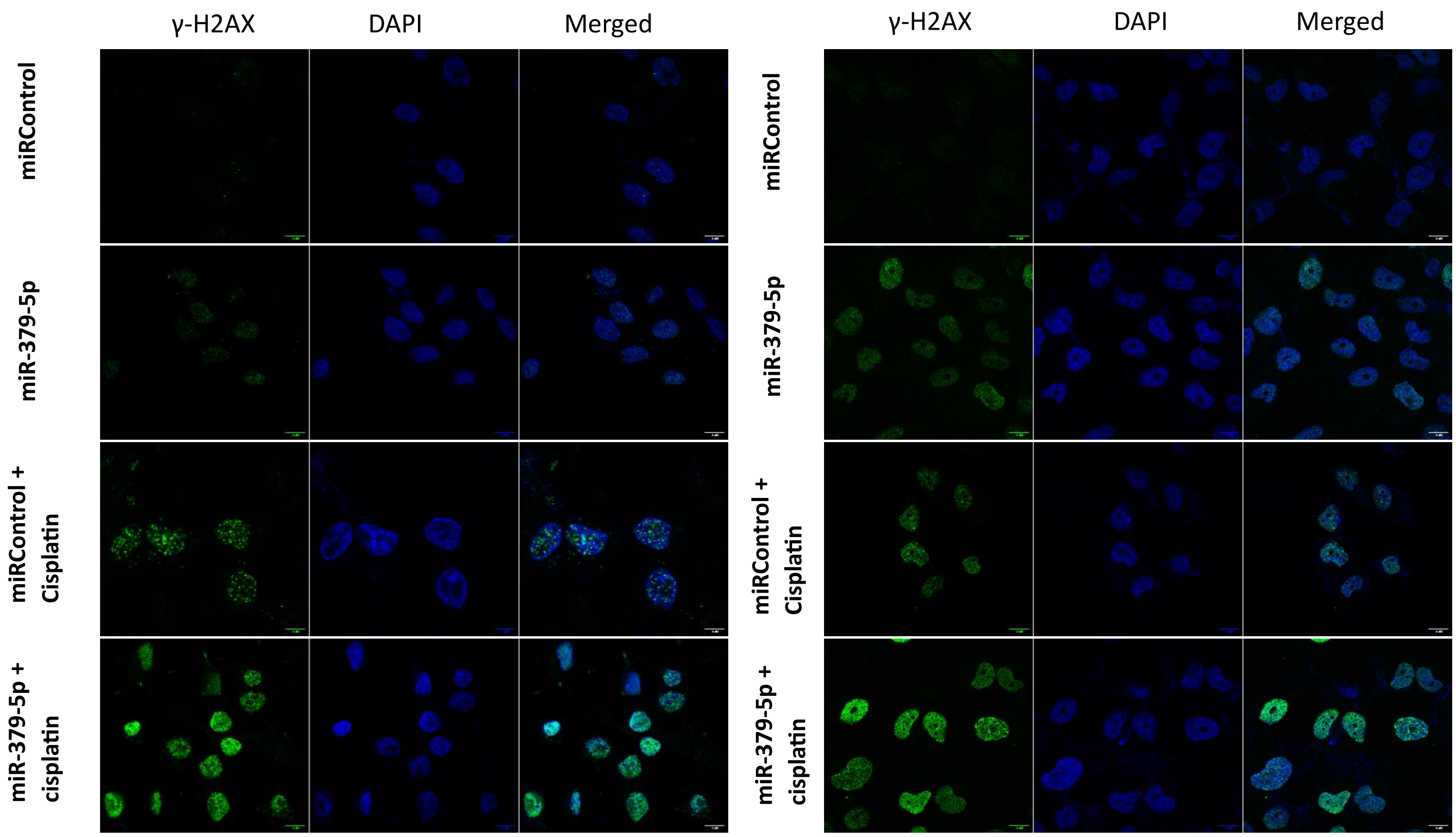

Fig. 9: γ-H2AX staining of SKOV3 and OVCAR3 cells. miRControl and miR-379-5p transfected cells were treated with either PBS or 10μM cisplatin for 12 hours. The images were acquired using a confocal microscope. Scale bar = 10μM.

Supplementary Fig. 10

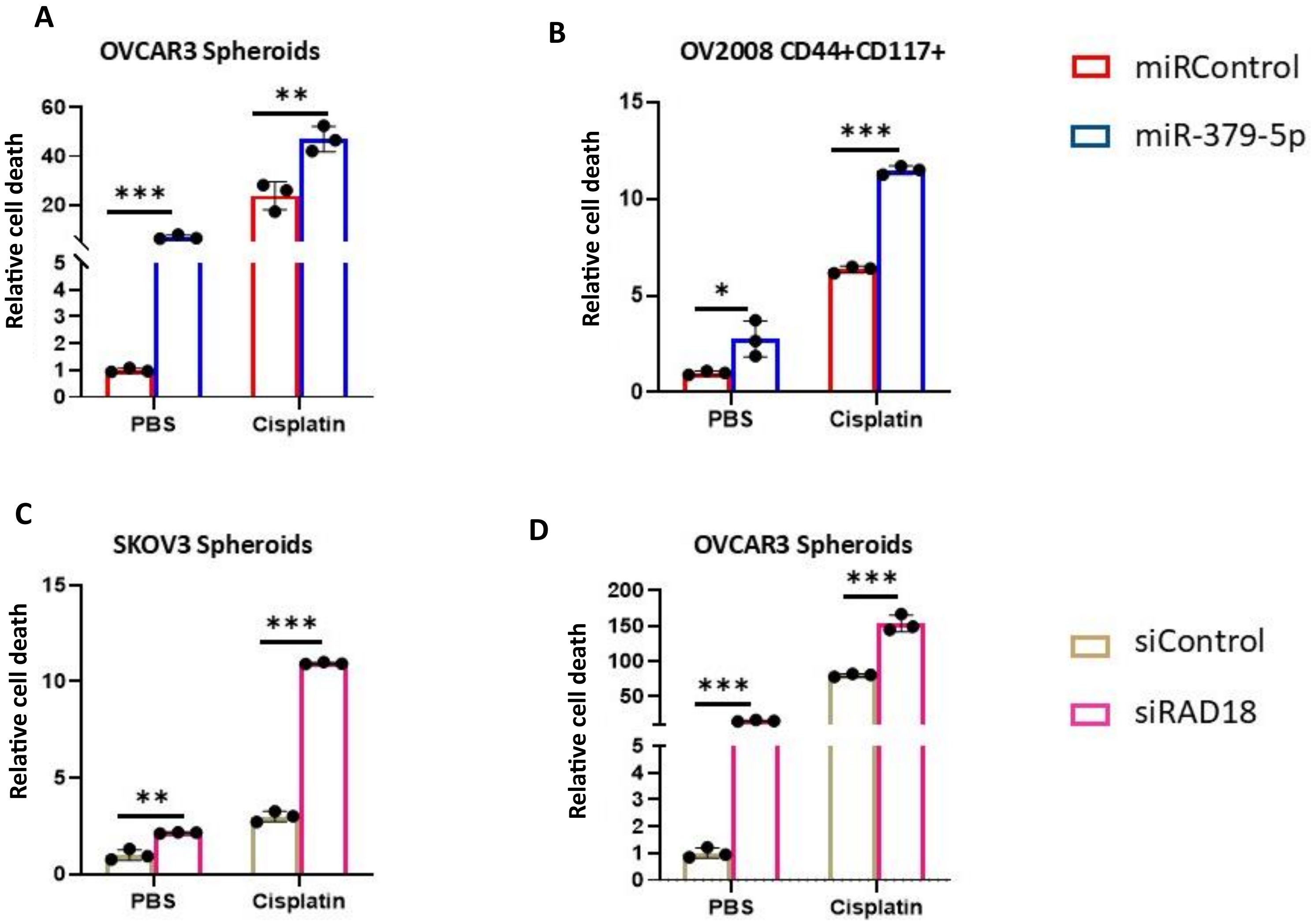

Fig. 10: Live Dead Assay on miR-379-5p transfected (A) OVCAR3 spheroids and (B) OV2008 CD44+CD117+ cells, and siRAD18 knockdown (C) SKOV3 spheroids and (D) OVCAR3 spheroids. The data are expressed as Relative Cell Death i.e. (miR-379-5p/ miRControl) and (siRAD18/ siControl). The graphs were represented as mean ± S.D., n = 3, Significance levels *p≤ 0.05, **p≤ 0.01 and ***p≤ 0.001.

1. Li R, Qu H, Wang S, Chater JM, Wang X, Cui Y, et al. CancerMIRNome: an interactive analysis and visualization database for miRNome profiles of human cancer. Nucleic Acids Res [Internet]. 2022 Jan 7;50(D1):D1139–46. Available from: https://doi.org/10.1093/nar/gkab784

2. Vlachos IS, Zagganas K, Paraskevopoulou MD, Georgakilas G, Karagkouni D, Vergoulis T, et al. DIANA-miRPath v3.0: deciphering microRNA function with experimental support. Nucleic Acids Res [Internet]. 2015 Jul 1;43(W1):W460–6. Available from: https://doi.org/10.1093/nar/gkv403

3. Sticht C, De La Torre C, Parveen A, Gretz N. miRWalk: An online resource for prediction of microRNA binding sites. PLoS One [Internet]. 2018 Oct 18;13(10):e0206239-. Available from: https://doi.org/10.1371/journal.pone.0206239

4. Vejnar CE, Zdobnov EM. miRmap: Comprehensive prediction of microRNA target repression strength. Nucleic Acids Res [Internet]. 2012 Dec 1;40(22):11673–83. Available from: https://doi.org/10.1093/nar/gks901

5. Rehmsmeier M, Steffen P, Höchsmann M, Giegerich R. Fast and effective prediction of microRNA/target duplexes. RNA. 2004 Oct;10(10):1507–17.

6. Tang Z, Kang B, Li C, Chen T, Zhang Z. GEPIA2: an enhanced web server for large-scale expression profiling and interactive analysis. Nucleic Acids Res [Internet]. 2019 Jul 2;47(W1):W556–60. Available from: https://doi.org/10.1093/nar/gkz430

7. Chandrashekar DS, Bashel B, Balasubramanya SAH, Creighton CJ, Ponce-Rodriguez I, Chakravarthi BVSK, et al. UALCAN: A Portal for Facilitating Tumor Subgroup Gene Expression and Survival Analyses. Neoplasia [Internet]. 2017;19(8):649–58. Available from: https://www.sciencedirect.com/science/article/pii/S1476558617301793

8. Chandrashekar DS, Karthikeyan SK, Korla PK, Patel H, Shovon AR, Athar M, et al. UALCAN: An update to the integrated cancer data analysis platform. Neoplasia [Internet]. 2022;25:18–27. Available from: https://www.sciencedirect.com/science/article/pii/S147655862200001X

9. Anaya J. OncoLnc: Linking TCGA survival data to mRNAs, miRNAs, and lncRNAs. PeerJ Comput Sci. 2016;2016(6).

10. Szklarczyk D, Kirsch R, Koutrouli M, Nastou K, Mehryary F, Hachilif R, et al. The STRING database in 2023: protein–protein association networks and functional enrichment analyses for any sequenced genome of interest. Nucleic Acids Res [Internet]. 2023 Jan 6;51(D1):D638–46. Available from: https://doi.org/10.1093/nar/gkac1000
